# One Stone, Three Birds: An Air and Interface Stable Argyrodite Solid Electrolyte with Multifunctional Nanoshells

**DOI:** 10.1002/advs.202304117

**Published:** 2023-09-26

**Authors:** Junwu Sang, Kecheng Pan, Bin Tang, Zhang Zhang, Yiyang Liu, Zhen Zhou

**Affiliations:** ^1^ Interdisciplinary Research Center for Sustainable Energy Science and Engineering (IRC4SE^2^) School of Chemical Engineering Zhengzhou University Zhengzhou 450001 P. R. China

**Keywords:** air stability, all‐solid‐state batteries, interface compatibility, Li_6_PS_5_Cl, solid electrolyte

## Abstract

Li_6_PS_5_Cl (LPSC) solid electrolytes, based on Argyrodite, have shown potential for developing high energy density and safe all‐solid‐state lithium metal batteries. However, challenges such as interfacial reactions, uneven Li deposition, and air instability remain unresolved. To address these issues, a simple and effective approach is proposed to design and prepare a solid electrolyte with unique structural features: Li_6_PS_4_Cl_0.75_‐OF_0.25_ (LPSC‐OF_0.25_) with protective LiF@Li_2_O nanoshells and F and O‐rich internal units. The LPSC‐OF_0.25_ electrolyte exhibits high ionic conductivity and the capability of “killing three birds with one stone” by improving the moist air tolerance, as well as the interface compatibility between the anode or cathode and the solid electrolyte. The improved performance is attributed to the peculiar morphology and the self‐generating and self‐healing interface coupling capability. When coupled with bare LiCoO_2_, the LPSC‐OF_0.25_ electrolyte enables stable operation under high cutoff voltage (≈4.65 V vs Li/Li^+^), thick cathodes (25 mg cm^−2^), and large current density (800 cycles at 2 mA cm^−2^). This rationally designed solid electrolyte offers promising prospects for solid‐state batteries with high energy and power density for future long‐range electric vehicles and aircrafts.

## Graphical abstract

A rationally designed Li_6_PS_5_Cl‐OF_0.25_ electrolyte incorporates protective LiF@Li_2_O nanoshells and F and O‐rich internal structure units, which enable simultaneous improvements in moist air tolerance, as well as compatibility at the Li/solid electrolyte and solid electrolyte/LiCoO_2_ interfaces.

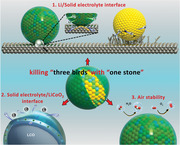

## 1 Introduction

All‐solid‐state lithium metal batteries (ASSLMBs) that utilize solid electrolytes (SEs) as replacement for flammable organic liquid electrolytes in Li‐ion batteries (LIBs) are highly promising candidates for next‐generation batteries with higher energy density and safety.^[^ ^1^, ^2^, ^3^ ^]^ Sulfide‐based SEs are known for comparable ionic conductivity to that of liquid electrolytes and are therefore considered as a potential solution for all‐solid‐state batteries.^[^ ^4^, ^5^, ^6^ ^]^ Moreover, sulfide‐based SEs possess acceptable mechanical strength, negligible grain boundary effects, and moderate cost.^[^ ^7^, ^8^, ^9^ ^]^ Therefore, sulfide‐based SEs, in particular, the argyrodite Li_6_PS_5_Cl (LPSC), are regarded as one of the most promising candidates for commercializing ASSLMBs. However, the development of LPSC electrolytes has been plagued by two major issues. The notorious instability at the electrode/SE interface deteriorates battery performance, and the air‐sensitive nature of LPSCs poses challenges to their practical manufacturing and application.^[^ ^10^, ^11^, ^12^ ^]^

The susceptibility of LPSC to reduction by Li metal and oxidation by cathode materials presents a challenge for ASSLMBs, leading to the formation of unstable and uneven interphases and battery degradation. Additionally, LPSC decomposes when its potential is reduced below 1.7 V or oxidized above 2.1 V versus Li/Li^+^, producing byproducts such as Li_2_S, Li_3_P, P, S, and LiCl.^[^ ^13^, ^14^, ^15^ ^]^ Although a few decomposition products of LPSC, such as LiCl, exhibit thermodynamic stability when in contact with Li metal, their low ionic conductivity and poor oxidation stability make them unsuitable for use in ASSLMBs. Artificial layers have been proposed to enhance the wettability and chemical stability between LPSC and electrodes; however, their high cost currently limits widespread use.^[^ ^16^, ^17^, ^18^, ^19^ ^]^ Moreover, artificial layers are unreliable in resisting lithium corrosion due to the large and inhomogeneous displacement of the interface, jeopardizing the mechanical integrity of the layer during cycling. Thus, there is a pressing need for a self‐generating passivating layer that can spontaneously form through the reaction of the SE with the electrodes. Such a layer would reform itself when the interphase is damaged, leading to improved performance and stability in ASSLMBs.

By introducing selective elements, it is possible to design solid electrolytes that can form specific products with desired functions.^[^ ^20^, ^21^, ^22^, ^23^ ^]^ Previous studies showed that the introduction of oxygen into LPSC can improve air stability and anodic interface stability by generating a large amount of P─O(─) bonds and conceivable passivation product Li_2_O.^[^ ^24^ ^]^ However, the electrochemical window of Li_2_O (0–3.1 V) is not sufficient for high‐voltage applications, while LiF (0–6.4 V) can be satisfied to match all current cathode materials for high‐voltage batteries.^[^ ^13^ ^]^ Moreover, the high bandgap of LiF (14.2 eV)^[^ ^25^ ^]^ and Li_2_O (8.37 eV)^[^ ^26^ ^]^ compared with LPSC (2.2 eV)^[^ ^27^ ^]^ ensures a perfect barrier for electron transfer. The decent bulk and shear modulus of LiF (66 and 45 GPa)^[^ ^28^ ^]^ and Li_2_O (85 and 67 GPa)^[^ ^29^ ^]^ are also favorable for suppressing Li dendrites and maintaining structural integrity. Therefore, LiF and/or Li_2_O were considered as potential partial precursor substitutes in the design of SEs. Passivating doping has shown to improve air stability (Li_2_O), anode compatibility (L_2_O and LiF), and cathode compatibility (LiF); however, they can also lead to reduced ionic conductivity due to the strong electrostatic interaction with Li^+^ ions (e.g., with F and O).

In addition, a special structural design allows core components to play a diversified role. For example, the LPSC‐based solid electrolyte with a special LiCl nanoshell constructed by Zeng et al. has demonstrated better anode interface stability meanwhile maintaining high ionic conductivity.^[^ ^30^ ^]^ However, the special structure still cannot solve the problem of awful air stability inherent in sulfide SEs. Thus, it is crucial and challenging to find a solid electrolyte with high ionic conductivity to simultaneously achieve high air stability and anode/SE/cathode interface compatibility.

Herein, we report the successful preparation of a high‐ionic‐conductivity LPSC‐OF_0.25_ electrolyte, which incorporates LiF@Li_2_O nanoshells and F/O‐rich internal units. This unique design (**One stone**) allows for air stability, as well as the compatibility for both anode/SE and SE/cathode interfaces (**Three birds**) synchronously. This special structure provides a new strategy for achieving high‐performance ASSLMBs.

## 2 Results and Discussion

### 2.1 “One Stone”: Design and Preparation of LPSC‐OF_0.25_ with Nanoshells

**Figure 1:**
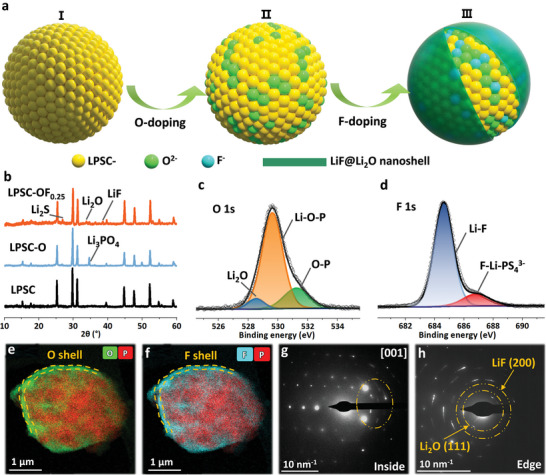
Structure design, preparation, and analyses of LPSC‐O_x_F_y_ argyrodites. a) Schematic diagram of the synthetic mechanism. b) XRD patterns of LPSC, LPSC‐O, and LPSC‐OF_0.25_ samples. b) XPS of LPSC‐OF_0.25_; c) O 1s and d) F 1s. The EDS images (e and f), and the SAED patterns for the inner (g) and edge (h) area of LPSC‐OF_0.25_.

A series of Li_6_PS_5‐x_Cl_1‐y_O_x_F_y_ (LPSC‐O_x_F_y_) solid electrolytes were prepared by using conventional solid‐state reactions with slow annealing treatment. The schematic design and preparation process of LPSC‐O_x_F_y_ is shown in **Figure** **1**a. The successful introduction of O into LPSC (**I**) with an equilibrium point (**II**) is crucial to the process. Additionally, based on the optimal O‐doping ratio, different proportions of fluorine doping are regulated to construct the LiF@Li_2_O nanoshell (**III**).

All the X‐ray diffraction (XRD) patterns of the prepared solid electrolytes exhibit a typical argyrodite structure (cubic phase, space group F$\bar{4}$3m, Figure S1a, Supporting Information).^[^ ^20^ ^]^ With increasing the O (or F) content, the main peak shifts toward a higher angle (Figure S1b, Supporting Information), indicating lattice distortion. The reduction of the lattice parameter results from the smaller ionic radius of O compared with that of S (F vs Cl) at the same Wyckoff position. The introduction of O and F doping may cause impurity segregation, leading to the generation of Li_2_S and Li_3_PO_4_ (Figure 1b).^[^ ^20^, ^24^ ^]^ Raman spectroscopy corroborates the trend of Li_2_S (374 cm^−1^) and Li_3_PO_4_ (950 cm^−1^) impurities observed in XRD (Figure S2, Supporting Information).^[^ ^24^ ^]^ When the content of O or F is further increased (e.g., x = 1.5, x = 1, and y = 0.25), the Li_2_O and LiF peaks appear due to the excessive precursor that cannot be stabilized in the lattice. Interestingly, introducing F into the optimal oxygen‐doped material Li_6_PS_4_OCl (LPSC‐O) results in the regeneration of Li_2_O and a decrease in the Li_3_PO_4_ content (Figure 1b). The introduced F partially replaces Cl, leading to further shrinkage of the unit cell, which will precipitate Li_2_O.

The X‐ray photoelectron spectroscopy (XPS) of O 1s and F 1s also confirms the presence of new O and F bonding in Li_6_PS_4_Cl_0.75_‐OF_0.25_ (LPSC‐OF_0.25_) (Figure 1c,d). The O 1s spectrum reveals peaks corresponding to Li_2_O (528.5 eV), Li‐O‐P (529.6 eV), and O‐P (531.2 eV), indicating the incorporation of O into the lattice and the existence of excessive precursor Li_2_O.^[^ ^31^, ^32^ ^]^ Similarly, the F 1s spectrum exhibits peaks at 684.8 and 686.9 eV, corresponding to the Li─F bond and the F─Li─PS_4_^3−^ bond, respectively.^[^ ^20^ ^]^ The peak intensity of PO_x_S_y_ (133.8 eV) is weakened due to the precipitation of Li_2_O (Figure S3f, Supporting Information), which is consistent with the XRD results.^[^ ^16^, ^32^ ^]^ Meanwhile, the intensity of PS_4_^3−^ (131.9 eV) remains the strongest (Figure S3f, Supporting Information), ensuring fast bulk Li^+^ transportation. Additionally, the Li─F bond results from not only F substitution in the crystal structure, but also the presence of extra LiF impurities.^[^ ^33^, ^34^ ^]^ The peak at 686.9 eV confirms the chemical interaction between F and PS_4_^3−^ in the lattice.^[^ ^20^ ^]^

Scanning transmission electron microscopy‐high‐angle annular dark field (STEM‐HAADF) and energy dispersive spectroscopy (EDS) images were used to confirm the generation of O and F nanoshells (≈80 nm) at the edge of the grain boundary in the as‐prepared LPSC‐OF_0.25_ (Figure 1e,f). Due to the detection limitation of EDS for Li, selected area electron diffraction (SAED) was used to analyze the phase distribution. In the inner region, the diffraction pattern shows a single crystal dot matrix of crystallized LPSC‐OF_0.25_, while a polycrystalline ring (within the yellow circle) is also present (Figure 1g). In contrast, the edge pattern is fundamentally different from the inner region, where only the polycrystalline diffraction rings of Li_2_O (111) and LiF (200) can be observed (Figure 1h). This strongly supports the formation of a LiF@Li_2_O nanoshell at the edge, which is rich in F and O. Furthermore, the results obtained by combining LPSC‐O (Figure S6, Supporting Information) and LPSC‐OF_0.15_ (Figure S7, Supporting Information) suggest that a small amount of doping O and F replace S and Cl within the lattice. When the concentration of light elements (O and F) becomes excessive, they are unable to remain stable within the lattice. However, during the slow cooling process, they become enriched in the surface of particles (Figure 1g,h).^[^ ^30^ ^]^ To sum up, the LPSC‐OF_0.25_ electrolyte possesses a LiF@Li_2_O nanoshell and F and O‐rich inner unit.

The Nyquist plots of LPSC‐O_x_F_y_ with different compositions (Figure S9a,b, Supporting Information) exhibit a nearly disappeared semicircle and a spike, which is attributed to ion hopping through the bulk and the blocking electrodes.^[^ ^35^ ^]^ This behavior is expected due to the high ionic conductivity. The ionic conductivity of pure LPSC (Figure S9c, Supporting Information) reaches 3.56 mS cm^−1^ at 25 °C, which is in good agreement with the published results.^[^ ^20^, ^30^ ^]^ However, for LPSC‐OF_0.25_ (Figure S9c, Supporting Information), the value decreases to 2.47 mS cm^−1^, but still surpasses the ionic conductivity of most fast ion conductors. This is mainly due to the fact that the PS_4_^3−^ bonds, which have low electrostatic force, still dominate, and the thin LiF@Li_2_O nanoshell (80 nm) allows for fast Li^+^ transport. Therefore, the highly ionic conductive LPSC‐OF_0.25_ electrolyte was successfully developed with a protective LiF@Li_2_O nanoshell and F and O‐rich inner unit.

### 2.2 “Bird I”: Tolerance against Humid Air

**Figure 2:**
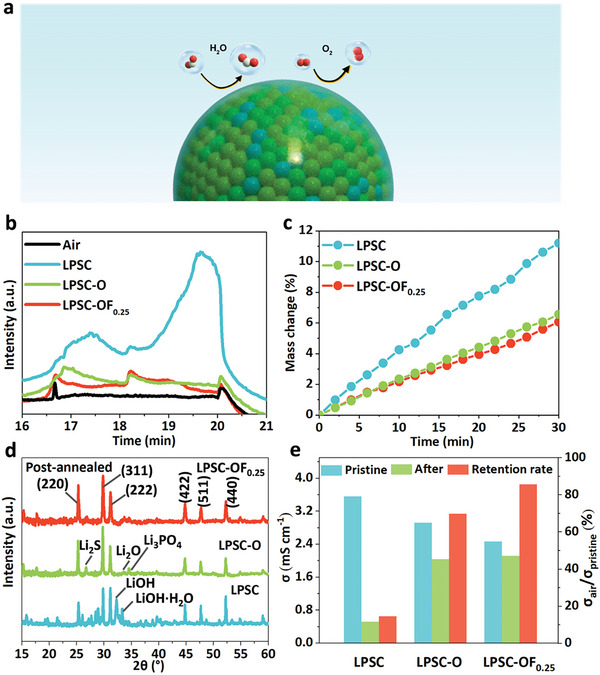
Verifying the tolerance against moist air. Test parameters: 0.5 h, relative humidity: 35 ± 2%, 25 °C. a) Mechanism of LPSC‐OF_0.25_ in humid air. b) HS‐GC tests for H_2_S yield of different samples after exposure to air. c) Mass changes for solid electrolytes as a function of time after exposure to air. d) XRD patterns of solid electrolytes with post annealing (150 °C, 2 h) after exposure to air. e) Ionic conductivity and restoration ratio of solid electrolytes after post annealing.

Sulfide‐based SEs are known to be susceptible to hydrolysis by moisture, which cannot only produce harmful H_2_S but also degrade their conductive frameworks.^[^ ^12^ ^]^ Moreover, sulfides tend to adsorb moisture or form hydrates easily.^[^ ^36^ ^]^ Here, the air stability of SEs was evaluated under atmospheric conditions (35 ± 2% humidity) and standard temperature and pressure, instead of the typically used low humidity (<20%) in most literature.^[^ ^37^, ^38^ ^]^ The headspace gas chromatography (HS‐GC) indicated that the successful incorporation of O can effectively suppress the generation of H_2_S (**Figure** **2**b). Furthermore, the mass change of LPSC‐OF_0.25_ over time upon exposure to air indicates a significantly lower proportion of adsorbed moisture compared with LPSC (Figure 2c).

To investigate the effect of moisture on the conductive framework, we conducted XRD before and after annealing. After heat treatment at 150 °C for 2 h, LPSC‐OF_0.25_ showed a complete recovery of its crystal structure (Figure 2d; Figure S10c, Supporting Information). In contrast, LPSC was unable to recover its crystal structure and had a certain amount of hydrates, as shown in Figure 2d and Figure S10a (Supporting Information). This indicates that moist air caused irreparable damage to LPSC and that the as‐crystallized water could not be completely removed by low‐temperature treatment. The structure of LPSC‐O mostly recovered with a slight increase in Li_2_S and Li_2_O due to the destruction of P─S bonds (Figure 2d; Figure S10b, Supporting Information).^[^ ^36^ ^]^ This suggests that the introduction of O can effectively inhibit the deterioration of the conductive framework by moisture.

As previously reported,^[^ ^12^ ^]^ LPSC is air‐sensitive, which increases the cost of product processing, preservation, and transportation. To further evaluate the superior air stability of our solid electrolyte, the ionic conductivity was tested for LPSC, LPSC‐O, and LPSC‐OF_0.25_ by electrochemical impedance spectroscopy (EIS) after air exposure and annealing (Figure 2e). LPSC‐OF_0.25_ showed the highest retention ratio (86%) with high σ_Li+_ (2.12 mS cm^−1^), while LPSC exhibited the lowest retention ratio (15%), followed by LPSC‐O (70%). Although LPSC‐O has concomitantly formed PO_x_S_y_ bonds that increase its air stability, the exposed PS_4_^3−^ can still be hydrolyzed by moisture. In contrast, the LiF@Li_2_O nanoshell of LPSC‐OF_0.25_ can effectively prevent moisture corrosion from entering the inner structure (Figure 2a). Therefore, LPSC‐OF_0.25_ can potentially resist structural changes under high humidity and shows favorable market competition compared with most sulfide‐based SEs (see details in Table S1, Supporting Information).

### 2.3 “Bird II”: Interfacial Compatibility against Li Metal

The critical current density (CCD) is an important parameter for evaluating the maximum current resistance without a short circuit.^[^ ^39^, ^40^ ^]^ LPSC‐OF_0.25_ exhibits a superhigh CCD of 2 mA cm^−2^, followed by 1.6 mA cm^−2^ of LPSC‐O, while the pristine LPSC only shows 1 mA cm^−2^, as disclosed in Figure S12 (Supporting Information). Additionally, LPSC‐OF_0.25_ demonstrates a specific areal capacity as high as 1 mAh cm^−2^. These excellent performances make it possible for solid‐state batteries to achieve higher mass loading, energy density, and power density.

The long‐term Li plating/stripping reversibility of the Li|SE|Li symmetric cells was tested at 60°C to evaluate the performance in terms of Li deposition behavior and degree of interfacial degradation, providing insight into the overall interfacial compatibility of Li|SE. At a current density of 0.2 mA cm^−2^, the voltage continuously increased before a sudden short circuit occurred in the Li|LPSC|Li cell, indicating severe interfacial degradation (Figure S13a, Supporting Information). In contrast, the degradation was milder in LPSC‐O and LPSC‐OF_0.25_, with the voltage curve almost flat.

The change in overpotential at the initial stage can be observed more clearly through the magnification of the voltage curve (Figure S13b, Supporting Information). In contrast to the continuously increasing voltage polarization in LPSC, the overpotential of LPSC‐O remains unchanged, while the overpotential of LPSC‐OF0.25 first slows down and then stabilizes. This may be due to the formation of an advantageous interphase after LPSC‐OF_0.25_ reacts with lithium metal, resulting in a higher Li^+^ migration rate over the original physical contact. Driven by chemical and electrochemical forces, the voltage reaches a steady state after a certain period. Moreover, the cycling life of LPSC‐OF_0.25_ (1600 h) is much longer than that of LPSC (543 h) and LPSC‐O (685 h), as well as other LPSC‐OF_y_, with details shown in Figures S13 and S14 (Supporting Information). This scenario confirms that the detrimental and continuing interfacial reactions of argyrodite‐based solid electrolytes are effectively inhibited by the formation of a LiF@Li_2_O nanoshell.

**Figure 3:**
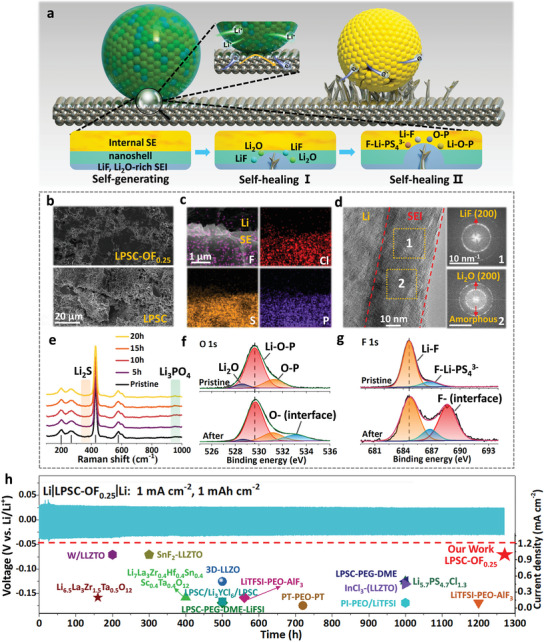
Investigating interfacial compatibility of LPSC‐O_x_F_y_ against Li by Li|SE|Li cells. a) Schematic diagram of interface compatibility between Li metal and LPSC‐OF_0.25_, or pristine LPSC. b) SEM of LPSC and LPSC‐OF_0.25_ surface after 200 h cycles. c) Cross‐sectional SEM and EDS of Li|LPSC‐OF_0.25_ interface after 200 h cycles. d) HRTEM and FFT of Li metal after 200 h cycles by Cryo‐TEM. e) In situ Raman spectra of Li|LPSC‐OF_0.25_. f,g) XPS of O 1s and F 1s at Li**|**LPSC‐OF_0.25_ interface after 200 h cycles. h) Long‐term Li plating/stripping cycling of Li|LPSC‐OF_0.25_|Li cells at 1 mA cm^−2^ with 1 mAh cm^−2^. The bottom half of the image is a histogram of current density and cycle time compared with the most advanced polymer, inorganic, and composite solid electrolytes; the details are shown in Table S2 (Supporting Information).

The self‐healing behavior of LPSC‐OF_0.25_ is thought to contribute to its superlong cycle life (**Figure** **3**a; Figure S13, Supporting Information). Although the overall voltage changes very little after reaching a steady state in the Li|LPSC‐OF_0.25_|Li cell, interesting phenomena were observed in several places. In some intervals (420–500, 700–730, and 1020–1040 h), the voltage dropped and then returned to the original state (Figure S13, Supporting Information). This voltage drop may be due to the rupture of the originally formed solid electrolyte interphase (SEI) caused by lithium dendrites, which shortens the counter‐electrode spacing. However, when the dendritic lithium recontacts with the remaining nanoshell or the internal structure, a new passivation interface is generated, which ensures the integrity of the protective interface. This interesting phenomenon is a classic example of self‐healing behavior.^[^ ^30^, ^41^, ^42^ ^]^ LPSC‐OF_0.25_ continues to self‐heal, forming a relatively stable passivation layer with lithium, which helps achieve an ultralong cycle life.

To explore the structure and composition of the SEI of different SEs, a series of characterizations were performed on the symmetric cells cycled for 200 h. LPSC shows an ever‐increasing interfacial resistance, which corresponds to the increased overpotential, as seen in Figure S15a (Supporting Information). In contrast, the interfacial resistance of LPSC‐O increases slowly, which indicates a relatively stable SEI, as shown in Figure S15b (Supporting Information). Interestingly, the interfacial resistance of LPSC‐OF_0.25_ decreases with cycling, corresponding to a continuously optimized interface that leads to a decrease in overpotential, as seen in Figure S15c (Supporting Information). The change in interfacial resistance indicates improved stability against Li, which is attributed to the presence of the LiF@Li_2_O nanoshell.

The morphology evolution was also compared among different electrolytes after cycling. The surface of LPSC‐OF_0.25_ is smooth, dense, and continuous, but in LPSC there are obvious cracks and holes (Figure 3b). This is probably due to the uneven interface between Li and LPSC, which leads to non‐uniform Li deposition and dendrite formation (Figures S16 and S17, Supporting Information). The uneven Li deposition and dendrite growth increase local stress, resulting in electrolyte fracture (i.e., short circuit).^[^ ^43^, ^44^, ^45^ ^]^ In contrast, LPSC‐OF_0.25_ maintains an intact and smooth electrolyte surface due to a stable and uniform SEI as well as a complete and flat interface with lithium metal (Figure 3b,c).^[^ ^46^, ^47^ ^]^

The Li|LPSC‐OF_0.25_|Li cell exhibits a uniform and thin SEI (35 nm) at the edge of lithium metal, as observed by a cryo‐transmission electron microscope (Cryo‐TEM) after cycling (Figure 3d). The interphase is mainly composed of crystallized LiF and Li_2_O, as well as partially amorphous compositions, as disclosed by the Fractional Fourier Transform (FFT) (Figure 3d). The crystalline LiF and Li_2_O with high bulk/shear modulus can effectively inhibit lithium dendrite growth, while the remaining amorphous part can prevent electron transport and provide channels for Li^+^ diffusion to some extent. Additionally, the nanoscale SEI is thin enough to ensure the rapid diffusion of Li^+^.

To further investigate the changes in the interfacial composition of LPSC‐OF_0.25_ during cycling, in situ Raman spectroscopy was used. The LPSC structure (as indicated by the gray line in the spectrum), Li_2_S, and Li_3_PO_4_ peaks remain unchanged for 20 h, demonstrating that the LiF@Li_2_O nanoshell can protect the internal electrolyte from damage (Figure 3e). The chemical state of O and F was analyzed by XPS. In the O 1s spectrum, the proportion of Li_2_O is significantly reduced compared with the original state, while the proportions of Li‐O‐P and O‐P increase, and a new peak appears at 533.2 eV (Figure 3f). The composition of the new peak with higher binding energy is likely to be a possible binding state with F and O‐rich SEI (such as F‐Li‐O‐). Similarly, a new peak at 688.3 eV appears in the F 1s spectrum (Figure 3g).

The LiF@L_i2_O nanoshell is compatible with metallic Li, and hence the shell‐interconnected LiF@Li_2_O frameworks, encapsulating the argyrodite grains, play a crucial role in improving the performance of the electrolyte. During cycling, the LiF@Li_2_O nanoshell would bond to the Li anode and form a LiF@Li_2_O‐dominated interphase layer. Similar behavior has been observed in a Li_7−x_PS_6−x_Cl_x_ electrolyte with LiCl shells.^[^ ^30^ ^]^ This uniform and thin LiF@Li_2_O‐rich interphase layer, in turn, further suppresses the decomposition of the argyrodite by metallic Li. The excellent electronically insulating properties, and decent bulk and shear moduli of LiF and Li_2_O, are favorable for further mitigating Li dendrites. However, after prolonged cycling, Li dendrites may penetrate the LiF@Li_2_O‐dominated interphase layer. The remaining nanoshell and F, O‐rich structure units enable the argyrodite core to react with the penetrated Li dendrites until they break off, known as the self‐healing effect.

Thus, the interfacial compatibility of LPSC‐OF_0.25_ with Li was further verified through a higher current density test (1 mA cm^−2^) (Figure 3h). Remarkably, LPSC‐OF_0.25_ showed no short circuit over 1270 h of cycling, and the voltage variation was almost negligible compared with the initial stage. To demonstrate the superiority of the design, we compared the results with those of other advanced solid electrolytes and found that LPSC‐OF_0.25_ exhibited the best stability against lithium metal (Table S2, Supporting Information). The LiF@Li_2_O nanoshell in the electrolyte contributed to the strongest dendrite suppression capability and the best long‐term cycling stability at high current density, highlighting the unparalleled advantages of LPSC‐OF_0.25_ in enabling high‐energy‐density and high‐power‐density ASSLMBs.

### 2.4 “Bird III”: Compatibility against LiCoO_2_

To investigate the compatibility of solid electrolytes with cathodes, ASSLBs were assembled with unmodified LCO. The cycling stability of the Li‐In||LCO cell was evaluated with LPSC, LPSC‐O, and LPSC‐OF_0.25_ at a current density of 0.5 mA cm^−2^ (Figure S20, Supporting Information). The results show that the cells with LPSC‐OF_0.25_ achieved the highest specific capacity (129 mAh g^−1^) and capacity retention rate (≈100%) after 100 cycles, compared with LPSC‐O (71 mAh g^−1^, 57.9%) and LPSC (31 mAh g^−1^, 26.5%). The charge‐discharge curve of LPSC shows that the capacity continues to decay and the charging voltage plateau continues to increase, indicating that it cannot form an effective cathode electrolyte interphase (CEI) with unmodified LCO (Figure S20b, Supporting Information). While LPSC‐O shows slower decay in the initial tens of cycles (Figure S20c, Supporting Information), and the capacity fades rapidly after a period of cycling, indicating that LCO is difficult to maintain stability with LPSC‐O. In contrast, LPSC‐OF_0.25_ demonstrated improved CEI stability in terms of capacity retention and voltage plateau stabilization (Figure S20d, Supporting Information). Overall, LPSC‐OF_0.25_ showed more efficient and stable CEI formation with unmodified LCO, resulting in a higher capacity and retention rate than LPSC and LPSC‐O.

**Figure 4:**
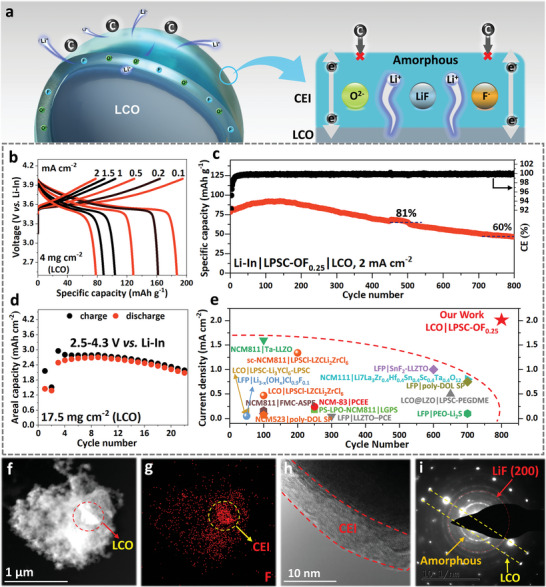
Demonstrating compatibility of LPSCl‐O_x_F_y_ against unmodified LCO by Li‐In|SE|LCO cells. a) Mechanism of CEI forming and structural features by LPSC‐OF_0.25_ and LCO. b) Charge/discharge profiles of Li‐In|LPSC‐OF_0.25_|LCO cells at different current densities within the voltage of 2.5–4 V (vs Li‐In). c) Discharge capacity and coulombic efficiency for Li‐In|LPSC‐OF_0.25_|LCO cells at 2 mAh cm^−2^ within the voltage of 2.5–4 V (vs Li‐In). d) Areal capacity for Li‐In|LPSC‐OF_0.25_|LCO cells with high loading cathodes (LCO: 17.5 mg cm^−2^) within the voltage of 2.5–4.3 V (vs Li‐In) after two cycles within the voltage of 2.5–3.9 V (vs Li‐In). e) Histogram of current density and cycle number, compared with the most advanced full cells with polymer, inorganic, and composite solid electrolytes matched with LFP, LCO, or NCM cathodes. f,g) STEM‐HAADF and EDS of F element of the composite cathode powder after cycles. h,i) HRTEM and SAED of the divested LCO after cycles.

The rate performance of the Li‐In|LPSC‐OF_0.25_|LCO full cell was evaluated under a higher cut‐off voltage (4.0 V vs Li‐In), and the results are shown in **Figure** **4**b and Figure S23 (Supporting Information). With high loaded voltage and fine contact by stacking pressure, LPSC‐OF_0.25_ displayed an impressive specific capacity of 187 mAh g^−1^ at 0.1 mA cm^−2^. After conversion (dividing the current density by the practical areal capacity to obtain the C‐rate), the specific capacities of 163 and 129 mAh g^−1^ were achieved at 0.2 mA cm^−2^ (≈1/3 C) and 0.5 mA cm^−2^ (1 C), respectively. Even at 2 mA cm^−2^ (≈7 C), a specific capacity of 79 mAh g^−1^ was still attained. Additionally, when the current density declined to 0.2 mA cm^−2^, the specific capacity recovered and remained stable. The initial Coulombic efficiency (CE) reached ≈80%, primarily due to the formation of CEI and SEI. The charge‐discharge curve for LPSC‐OF_0.25_ was stable and smooth under different current densities, indicating the absence of obvious side reactions (Figure 4b). Furthermore, the high reversibility of the cell suggests minimal loss of active substances (i.e., Li^+^).

To evaluate the long‐term cycling performance of LPSC‐OF_0.25_ at higher current density, the cell was tested at 2 mA cm^−2^ from start to finish. After slight attenuation, the capacity transitions into growth and maintains stability (Figure 4c). The cell can maintain high capacity and continuous stable operation even after 500 (81%) or 800 (60%) cycles, further demonstrating that the stable CEI and SEI formed ensure long‐term operation under ultra‐high current density. When compared with the latest full cells coupled with LiFePO_4_ (LFP), LCO, or LiNi_x_Co_y_Mn_z_O_2_ (NCM, x+y+z = 1) cathodes, the LPSC‐OF_0.25_|LCO full cell achieved the longest cycling under such a high current density (Figure 4e and Table S3, Supporting Information).

To verify the LPSC‐OF_0.25_^′^s ultrahigh voltage tolerance, the cutoff voltage was set from 4 to 4.3 V versus Li‐In. When considering the typical LCO behavior during discharge, the change of the discharge curve to steep slope is ≈3.8 V versus Li/Li^+^ while the value in Figure S24 (Supporting Information) is ≈3.45 V versus Li‐In, so we can translate to an actual charging cutoff potential of ≈4.65 V versus Li/Li^+^. Under 0.5 mA cm^−2^, the specific capacity increased from 136 to 163 mAh g^−1^, achieving 20% increase (Figure S24, Supporting Information). However, as the cutoff voltage increased, the CE showed a slight decline. In order to achieve high energy density in ASSLMBs, a thick cathode is also necessary, and an ultrathick cathode with a mass loading of 25 mg cm^−2^ (LCO: 17.5 mg cm^−2^) was constructed with a cutoff voltage of 2.5–4.3 V versus Li‐In. The initial two cycles were set to achieve low‐voltage activation (2.5–3.9 V vs Li‐In). After that, the cell achieved a high areal capacity of 2.7 mAh cm^−2^ (155 mAh g^−1^) and maintained a high areal capacity of 2.1 mAh cm^−2^ after 20 cycles (Figure 4d). The LPSC‐OF_0.25_ cell exhibited outstanding rate performance, excellent cycle stability, and stable operation under ultra‐high voltage and thick cathodes, demonstrating excellent cathode compatibility.

To gain insight into the internal mechanism of LPSC‐OF_0.25_, we analyzed the morphology and element distribution of the cathode after cycling. EDS images of the C element show that the conductive agent was uniformly distributed among the cathode, providing effective electron transport channels (Figure S25a, Supporting Information). By using STEM‐HADDF and EDS, we located the position of LCO through Co distribution (Figure 4f; Figure S25b, Supporting Information). Interestingly, F exhibits obvious aggregation covering LCO (Figure 4g), whereas P, S, and Cl do not (Figure S25c–e, Supporting Information). Considering the characteristics of LPSC‐OF_0.25_, F is a high‐content element in the nanoshell and forms a stable CEI with LCO, effectively protecting the internal solid electrolyte. Moreover, O also exhibits similar aggregation and behavior as F, but it cannot be distinguished from O originating from LCO (Figure S25f, Supporting Information).

To remove the interference of the solid electrolyte and conductive agent, LCO was exfoliated, and only clear Co, O, and F elements were observed (Figure S26, Supporting Information). An ultrathin CEI with a thickness of 6–8 nm was demonstrated at the edges of the LCO (Figure 4h). The CEI was found to be mainly composed of a mixture of crystalline and amorphous phases. The composition was determined by nano‐SAED on the divested LCO edge (Figure S27, Supporting Information). To exclude the interference of the LCO phase, SAED was first performed on pristine LCO, and a clear pattern of a single crystal lattice was observed (Figure S28, Supporting Information). In the recycled LCO, the LCO lattice was still dominant, and additional diffraction patterns also appeared (Figure 4i). A halo diffusing outward was observed, indicating the formation of an amorphous phase (e.g., amorphous F‐Li‐O‐, similar to SEI) at the interface. Interference from amorphous conductive agents could be excluded (Figure S26, Supporting Information). The polycrystalline ring was primarily composed of LiF (200) crystal planes.

The formation of the LiF@Li_2_O nanoshell at the LPSC‐OF_0.25_|LCO interface is similar to that of SEI, leading to the construction of a uniform and dense CEI (Figure 4a). The LiF@Li_2_O‐dominated CEI protects the inner LPSC from decomposition at high current density and voltage, and its thin thickness (6–8 nm) facilitates fast ion‐electron transport through quantum tunneling, resulting in higher capacity at high rates.^[^ ^48^ ^]^ Moreover, the crystallized components of the CEI exhibit a wide electrochemical window (e.g., LiF is 0–6.4 V), reducing the decomposition of argyrodite and ensuring intact contact under high voltage. The uniformly dispersed conductive agents and solid electrolytes enable normal operation under thick cathodes, and the excellent compatibility between LPSC‐OF_0.25_ and unmodified LCO results in stable operation at high rates, high voltage, and thick cathodes.

## 3 Conclusion

The optimal structure of LPSC‐OF_0.25_ with its protective LiF@Li_2_O nanoshell and F and O‐rich inner units has been determined through a schematic design, experimental preparation, and performance verification. This design offers a “one stone killing three birds” solution by improving moist air tolerance, anode and cathode interface compatibility, and stability. The Li_2_O‐rich nanoshell of LPSC‐OF_0.25_ effectively prevents electrolyte corrosion under high humidity, providing an excellent retention ratio (86%) and high ionic conductivity (2.12 mS cm^−1^). LPSC‐OF_0.25_ also prevents the formation of lithium dendrites, and through its special structure and the coupling mechanism of spontaneous generation and self‐healing SEI, demonstrates the highest CCD (2 mA cm^−2^) and excellent cycling stability (>1200 h at 1 mA cm^−2^). Lastly, the self‐generated ultrathin and dense LiF and Li_2_O‐rich interphase allows the Li‐In|LPSC‐OF_0.25_|LCO cell to achieve stable operation at ultrahigh voltage (≈4.65 V vs Li/Li^+^) and thick cathodes (25 mg cm^−2^), achieving over 800 stable cycles at 2 mA cm^−2^. This new‐type solid electrolyte with high ionic conductivity can simultaneously improve air stability, and anode and cathode compatibility perfectly, thus showing tremendous potential for practical applications in high‐energy/power‐density ASSLMBs.

## 4 Experimental Section

### Materials Preparation

To prepare argyrodite solid electrolytes, the Li_2_O (Aladdin, 99.9%), Li_2_S (Macklin, 99.9%), LiF (Aladdin, 99.9%), LiCl (Aladdin, 99.9%), and P_2_S_5_ (Aladdin, 99.9%) powders were weighted with a total mass of 2 g according to the stoichiometric molar ratio of Li_6_PS_5−x_Cl_1‐y_‐O_x_F_y_ (x = 0, 0.5, 1, 1.5, y=0, and x=1, y = 0.05, 0.15, 0.25, and 0.35) and mixed in an agate mortar for 15 min. The powders were subjected to a ball milling (All‐directional planetary ball mill) with balls (zirconia) in the diameter of 10 mm for 2 h premixing at 200 rpm and then for 8 h milling at 500 rpm for mechanochemical synthesis. The resultant powders were pressed into pellets and vacuum sealed in a quartz tube for annealing at 530 °C for 8 h with a ramping rate of 1 °C min^−1^ and then with a cooling rate of 1 °C min^−1^ to 20 °C. The heat‐treated pellets were hand‐ground in an agate mortar for further use. Different contents of Li_2_O were employed as one of the raw materials in replacement of Li_2_S to prepare a series of Li_6_PS_5‐x_Cl‐O_x_ solid electrolytes (x = 0, 0.5, 1, and 1.5, designated as LPSC, LPSC‐O_0.5_, LPSC‐O, and LPSC‐O_1.5_, correspondingly). Furthermore, LiF was also employed as the raw material in replacement of LiCl to prepare Li_6_PS_4_Cl_1‐y_‐OF_y_ solid electrolytes (x = 1, y = 0.05, 0.15, 0.25, and 0.35, designated as LPSC‐OF_0.05_, LPSC‐OF_0.15_, LPSC‐OF_0.25_, and LPSC‐OF_0.35_, correspondingly). The universal type Li_6_PS_5‐x_Cl_1‐y_‐O_x_F_y_ solid electrolytes are abbreviated as LPSC‐O_x_F_y_. The samples were protected in an argon atmosphere (H_2_O and O_2_ ≤ 1 ppm) throughout all procedures.

### Materials Characterization

XRD was conducted under a Rigaku Ultima IV system (Cu Kα) with a scan rate of 5° min^−1^ within a 2θ range of 10°–60°. To prevent moisture exposure, the powders were sealed on a sample holder with polyimide films in an Ar glove box (H_2_O and O_2_ ≤ 1 ppm). For in situ Raman scattering tests, a LabRAM HR Evolution instrument with a 532 nm excitation source, and a home‐made spectro‐electrochemical cell obtained from Beijing Scistar Technology Co., Ltd. The cell was sealed with fluorine gaskets between the cell body and a transparent quartz window (10 mm) to enable laser beam focusing on the lateral surface of the battery pellet (Figure S18, Supporting Information). HS‐GC was performed on a Shimadzu GC‐2014C with a 10 °C min^−1^ increase in temperature after holding the target gas at 60 °C for 2 min. Before the HS‐GC test, the sample (20 mg) was placed in a closed and air‐rich glass bottle (20 mL), and after standing for 0.5 h, a fixed amount of gas was extracted for tests. XPS was conducted under an AXIS Supra facility. Surface and cross‐section morphology were observed with SEM (ZEISS SIGMA 500) equipped with EDS. TEM was conducted under FEI TalosF200S at 200 kV and Thermoscientific/Krios G3i at 300 kV. The TEM grid was loaded onto the cryo‐holder in an Ar atmosphere and quickly transferred to the TEM at a cryogenic temperature. To test the lithium metal interface after cycling, a symmetric cell was fabricated by loading the TEM grid (copper screen) and lithium metal, and the TEM grid was stripped for testing. For ex situ measurements (XPS, SEM, and EDS) of the interface, the specimens were collected from the Li|SE interface layer after detaching the cycled Li||Li symmetric cells in an Ar‐filled glove box (H_2_O and O_2_ ≤ 1 ppm). The specimens were loaded into an air‐tight sample holder and then transported from the Ar‐filled glovebox to the equipment used for the ex situ measurements.

### Electrochemical Measurements

EIS measurements were conducted by using a VMP‐300 electrochemical workstation over a frequency range from 0.1 Hz to 1 MHz. The ionic conductivity was assessed on SS|SE|SS (stainless steel) symmetric cells under an external pressure of 360 MPa. Galvanostatic measurements for critical current density (CCD) and Li plating/stripping were conducted on Li|SE|Li symmetric cells at 60 °C by using a LAND cell tester. The CCD was tested on Li symmetric cells by incrementing the current densities in steps of 0.1 mA cm^−2^ between 0.1 and 1 mA cm^−2^, followed by 0.2 mA cm^−2^ after 1 mA cm^−2^. Pellets for Li|SE|Li symmetric cells, CCD, and Li plating/stripping cycling were cold‐pressed from powders at 360 MPa. For in situ Raman spectroscopy measurements, the Li|SE|Li battery pellet (10 mm) was pressed through the aforementioned process and then placed into a home‐made spectro‐electrochemical cell (Figure S18, Supporting Information) and cycled at 60 °C under a current density of 0.2 mA cm^−2^ under the LAND cell tester. Finally, all symmetric cells were mounted on a coin cell.

### All‐Solid‐State Cell Assembly and Tests

All‐solid‐state cells were assembled in an Ar‐filled glove box by using unmodified LCO and Li–In as electrode materials. The single electrolyte was used as both the electrolyte diaphragm and cathode ion conductive additives. The Li–In electrode was prepared by manually rolling a piece of Li with a piece of In with a weight ratio of 7.5:92.5 with a stainless steel rod, and then cut into disks with a suitable diameter. For preparing the cathode, unmodified LCO was used as the cathode active material, and the corresponding electrolyte (same as the diaphragm electrolyte used) was adopted as the ionic conductor, together with Super P as the electron conductor. These were mixed manually in a mortar for 30 min. with a mass ratio of 7:2.5:0.5. Bilayer pellets containing the cathode (if not otherwise stated, LCO active material: 4 ± 0.5 mg cm^−2^) and solid electrolyte (100 mg cm^−2^) were pressed at ≈360 MPa, and then Li–In foil was attached to the electrolyte side. A specific pressurized cell mold (Figure S19, Supporting Information) was used under an external pressure of 150 MPa. Galvanostatic charging/discharging of the cells was performed at 60 °C under cut‐off voltages of 2.5–4 V and 2.5–4.3 V (vs Li–In). For the high‐loading cell test, the initial two cycles were set to achieve low‐voltage activation (2.5–3.9 V vs Li‐In). The battery assembling was performed in an Ar‐filled glove box (H_2_O and O_2_ ≤ 1 ppm). To accurately obtain a more realistic rate performance, the current density (mA cm^−2^) divided by the areal capacity (mAh cm^−2^) to obtain the C‐rate (h^−1^) was used.

### Statistical Analysis

The reproducibility was confirmed by measuring the electrochemical data multiple times to obtain an average value. The TEM and Cryo‐TEM images were analyzed on Digital Micrography software (Gatan Inc Pleasanton, CA, USA). The XRD, XPS, and Raman were analyzed on Jade (Materials Data, Livermore, CA, USA), CasaXPS (Casa Software Ltd.), and Labspec (Horiba, Kyoto, Japan) software, respectively. The Origin software was used for data analyses and processing. Other details have been included in the Methods, electrochemical tests, and chart annotation.

## Conflict of Interest

The authors declare no conflict of interest.

## Supporting information

Supporting Information

## Data Availability

The data that support the findings of this study are available on request from the corresponding author. The data are not publicly available due to privacy or ethical restrictions.
