# Supplementary material for: One Stone, Three Birds: An Air and Interface Stable Argyrodite Solid Electrolyte with Multifunctional Nanoshells
Source: Adv Sci (Weinh). 2023 Sep 26;10(32):2304117. doi: 10.1002/advs.202304117 (PMC10646260; doi:10.1002/advs.202304117)
Supplement: Supplementary file 1 — Supporting Information [file ADVS-10-2304117-s001.pdf]

ADVANCED SCIENCE
Open Access

Supporting Information

for *Adv. Sci.*, DOI 10.1002/advs.202304117

One Stone, Three Birds: An Air and Interface Stable Argyrodite Solid Electrolyte with Multifunctional Nanoshells

Junwu Sang, Kecheng Pan, Bin Tang, Zhang Zhang, Yiyang Liu and Zhen Zhou**

Supporting Information

One Stone, Three Birds: An Air and Interface Stable Argyrodite Solid Electrolyte with Multifunctional Nanoshells

Junwu Sang, Kecheng Pan, Bin Tang, * *Zhang Zhang, Yiyang Liu, Zhen Zhou* *

Interdisciplinary Research Center for Sustainable Energy Science and Engineering (IRC4SE^2), School of Chemical Engineering, Zhengzhou University, Zhengzhou 450001, PR China

*Corresponding author. Email: tangbin@zzu.edu.cn; zhenzhou@zzu.edu.cn

List directory

1. Characterizations of LPSC-O_xF_y electrolytes.

Figure S1. X-ray diffraction (XRD) patterns of annealed samples.
Figure S2. Raman spectroscopy of annealed samples.
Figure S3. XPS of annealed samples.
Figure S4. STEM-HADDF and EDS images of LPSC-$OF_{0.25}$.
Figure S5. Cry-TEM images and the corresponding SAED patterns of (a, b) LPSC, (c, d) LPSC-O and (e) LPSC-$OF_{0.25}$.
Figure S6. STEM-HADDF and EDS images of LPSC-O.
Figure S7. STEM-HADDF and EDS images of LPSC-$OF_{0.15}$.
Figure S8. STEM-HADDF and EDS images of LPSC-$OF_{0.35}$.
Figure S9. EIS of selected frequencies for measurements in SS|SE|SS cells at 25°C.

2. Verifying tolerance of LPSC-O_xF_y against humid air.

Figure S10. Contrast XRD patterns of pristine and post-annealed samples after exposure to air.
Figure S11. EIS of solid electrolytes after post-annealing.
Table S1. Air stability of LPSC-O_xF_y and previously reported solid electrolytes.

3. Investigating interfacial compatibility of LPSC-O_xF_y against Li metal.

Figure S12. Voltage (solid lines) and current (dotted lines) curves with time for LPSC, LPSC-O and LPSC-$OF_{0.25}$.

Figure S13. Long-term Li plating/stripping cycling at 0.2 mA cm^{-2} with 0.1 mAh cm^{-2} for LPSC, LPSC-O and LPSC-$OF_{0.25}$.

Figure S14. Long-term Li plating/stripping cycling at 0.2 mA cm^{-2} with 0.1 mAh cm^{-2} for LPSC-$OF_{0.05}$, LPSC-$OF_{0.15}$ and LPSC-$OF_{0.35}$.

Figure S15. In-situ EIS measurements on the Li symmetric cells with (a) LPSC, (b) LPSC-O, or (d) LPSC-$OF_{0.25}$ as solid electrolytes.

Figure S16. SEM: (b) LPSC, (c) LPSC-O, and (d) LPSC-$OF_{0.25}$ surface and the corresponding EDS of Cl element after cycles.

Figure S17. Cross-sectional SEM and EDS images for the Li|LPSC interface after cycles.

Figure S18. (a) Drawing of the in-situ Raman mold. (b) Image of laser focusing area on the Li|LPSC-$OF_{0.25}$ interface.

Table S2. Stability of LPSC-$OF_{0.25}$ to lithium metal compared with the most advanced previously reported polymer, inorganic, and composite solid electrolytes.

4. Demonstrating compatibility of LPSCl-O_xF_y against unmodified LCO.

Figure S19. Schematic diagram of a pressurized cell.

Figure S20. (a) Discharge capacity and coulombic efficiency, and (b-e) corresponding charge/discharge profiles at 0.5 mAh cm^{-2} within the voltage of 2.5-4 V (*vs.* Li-In).

Figure S21. Charge/discharge curves at 0.5 mAh cm^{-2} within the voltage range of 2.5-4 V (*vs.* Li-In) for Li-In|SE|LCO cells with LPSC-$OF_{0.25}$ as the solid electrolyte and ion conductive additive in composite cathodes (25°C).

Figure S22. (a) Rate performance for Li|LPSC-$OF_{0.25}$|LCO cells within the voltage of 2.5-4.3 V (10 MPa). (b) Charge/discharge profiles at 0.1 mAh cm^{-2} within the voltage of 2.5-4.3 V for Li|LPSC-$OF_{0.25}$|LCO cells (50 MPa).

Figure S23. Rate performance and Coulombic efficiency for Li-In|LPSC-$OF_{0.25}$|LCO cells within the voltage of 2.5-4 V (*vs.* Li-In).

Figure S24. Charge/discharge profiles for Li-In|LPSC-$OF_{0.25}$|LCO cells within different voltage ranges (2.5-4 V and 2.5-4.3 V *vs.* Li-In) under 0.5 mA cm^{-2}.

Figure S25. EDS images of the as-prepared composite cathode powder after 50 cycles (with LPSC-$OF_{0.25}$ as the solid electrolyte and ion conductive additive in composite cathodes).

Figure S26. STEM-HAADF and EDS images of the divested LCO after 50 cycles (with LPSC-$OF_{0.25}$ as the solid electrolyte and ion conductive additive in composite cathodes).

Figure S27. Cry-TEM image of the divested LCO from composite cathodes after 50 cycles (with LPSC-$OF_{0.25}$ as the solid electrolyte and ion conductive additive in composite cathodes).

Figure S28. SAED image of the pristine LCO.

Table S3. Cycling stability of LPSC-$OF_{0.25}$|LCO cells compared with the most advanced previously reported full cells with polymer, inorganic, and composite solid electrolytes matched with LFP, LCO, or NCM cathodes.

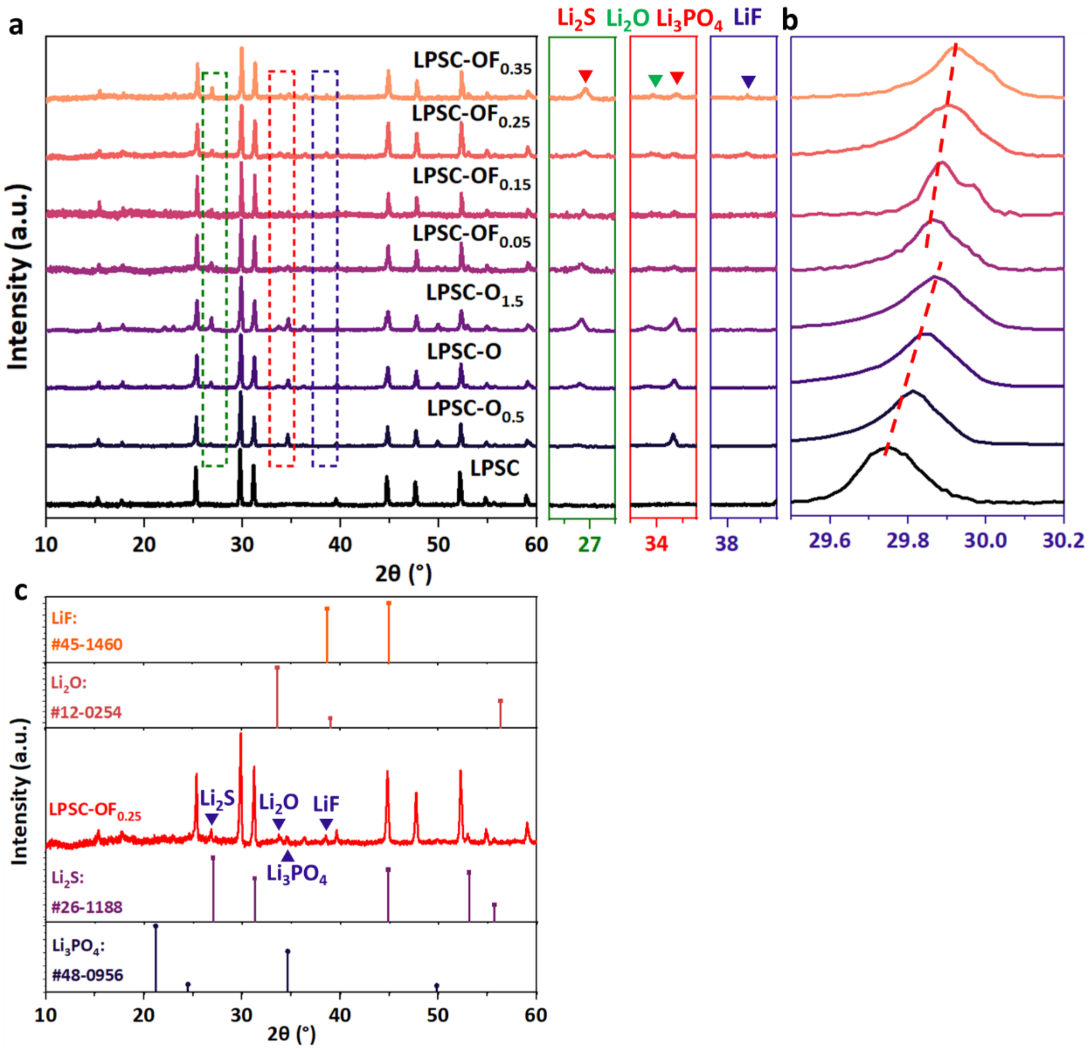

Figure S1. XRD patterns of annealed samples. (a) XRD patterns of LPSC-O_xF_y. All obtained XRD data were normalized with the strongest peak at 29.7°, and the ratio of other peaks to the strongest peak represents the ratio of relative content to a certain extent, so the change of the relative content can be obtained by the change of peak intensity. Meanwhile the substrate was removed from the patterns caused by the halos at low angles (10−30°), which are mainly caused by the polyimide film used to prevent the air. The polyimide film leads to the interference of the strongest peak (~21°) of Li_3PO_4, so we chose the second-strongest peak (~34.5°) for comparison. (b) Partial enlarged drawing. (c) XRD patterns of LPSC-$OF_{0.25}$ and standard XRD patterns of related impurities, Li_2S (PDF No. 26-1188), Li_2O (PDF No. 12-0254), Li_3PO_4, (PDF No. 48-0956) and LiF (PDF No. 45-1460). The LPSC-peak leads to the interference of the strongest peak (~45°) of LiF, so we chose the second-strongest peak (~38.7°) for analyses.

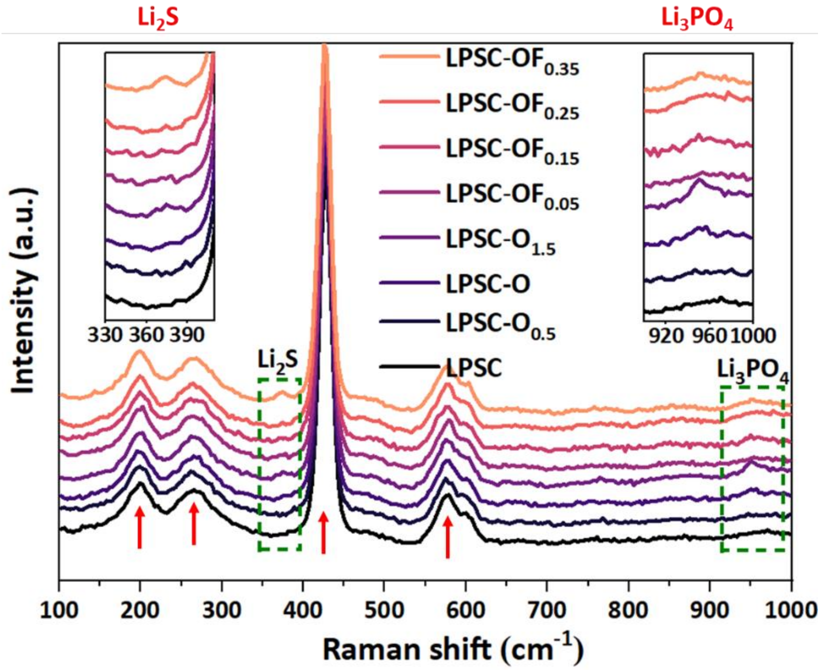

Figure S2. Raman spectroscopy of annealed samples. Including LPSC (red arrows correspond to peaks), Li_2S and Li_3PO_4.

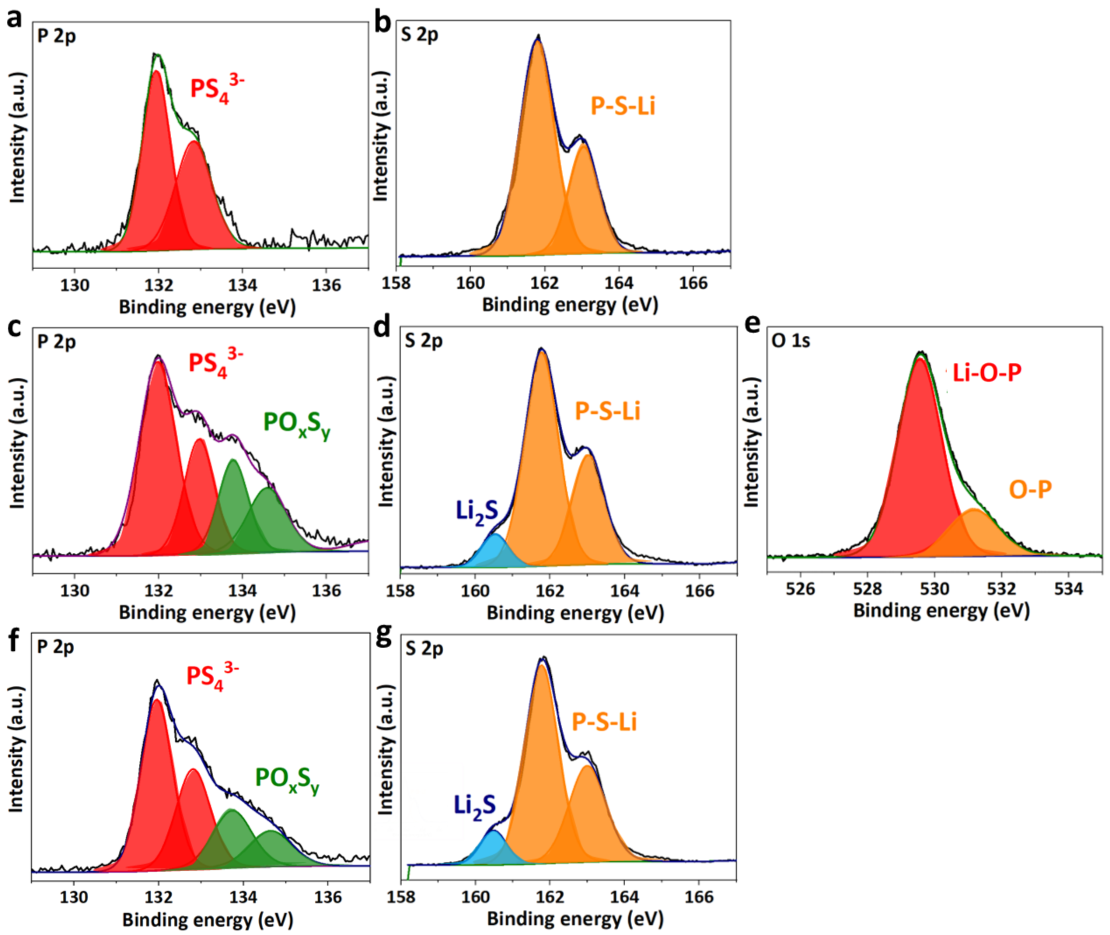

Figure S3. XPS of annealed samples. (a) P 2p and (b) S 2p of LPSC; (c) P 2p (d) S 2p and (e) O 1s of LPSC-O; (f) P 2p and (g) S 2p of LPSC-$OF_{0.25}$.

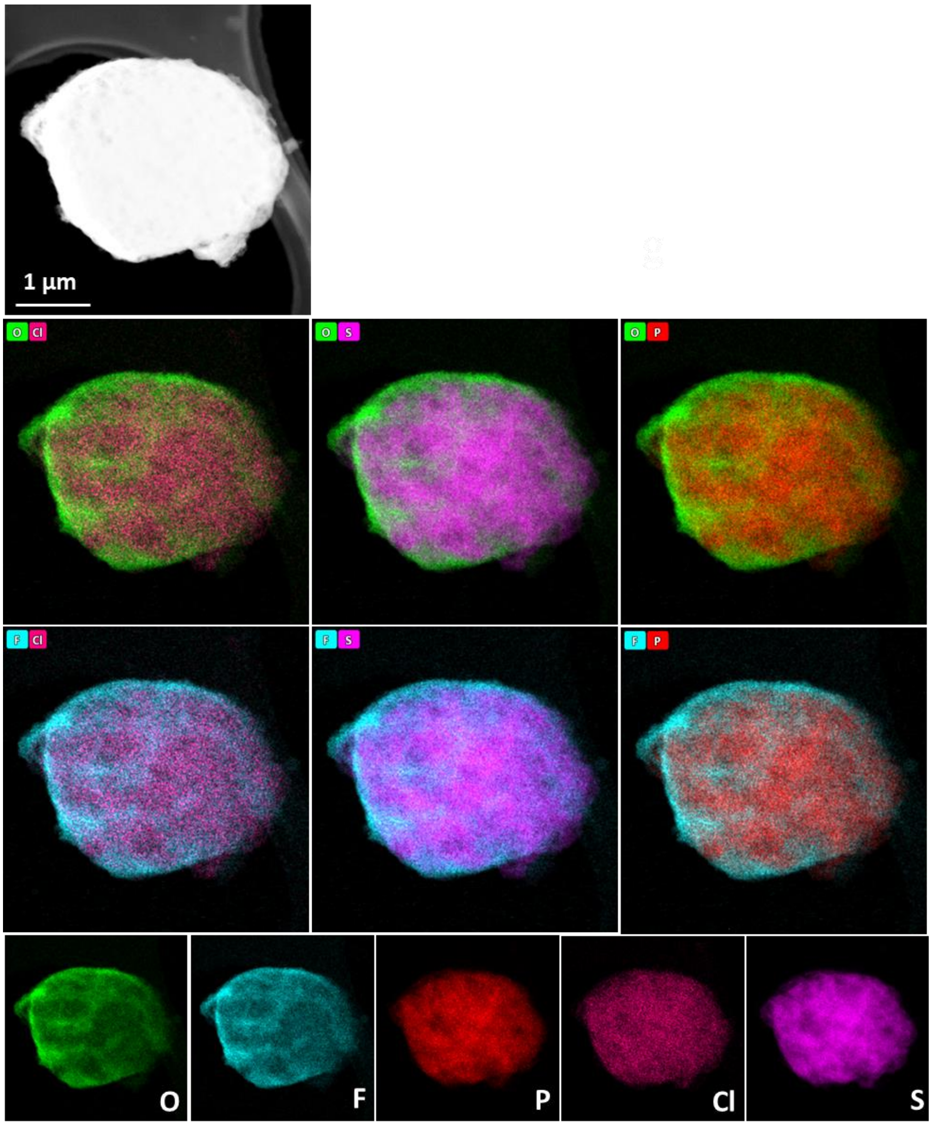

Figure S4. STEM-HADDF and EDS images of LPSC-$OF_{0.25}$.

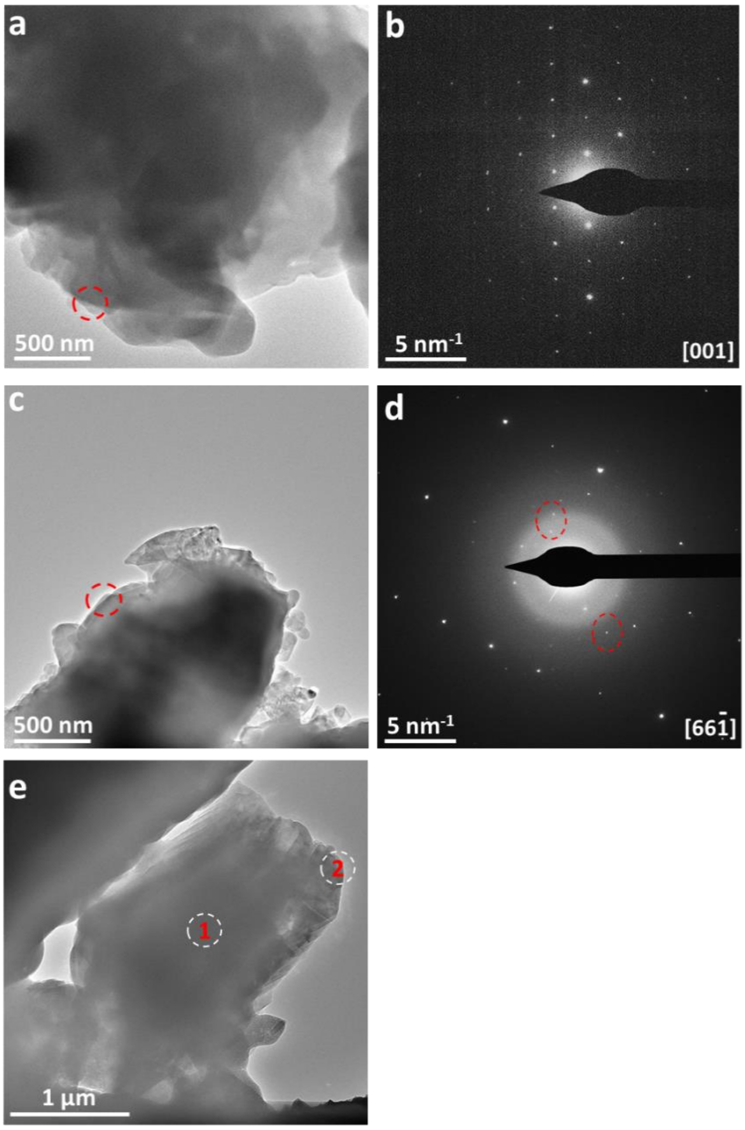

Figure S5. Cry-TEM images and the corresponding SAED patterns of (a, b) LPSC, (c, d) LPSC-O and (e) LPSC-OF$_{0.25}$.

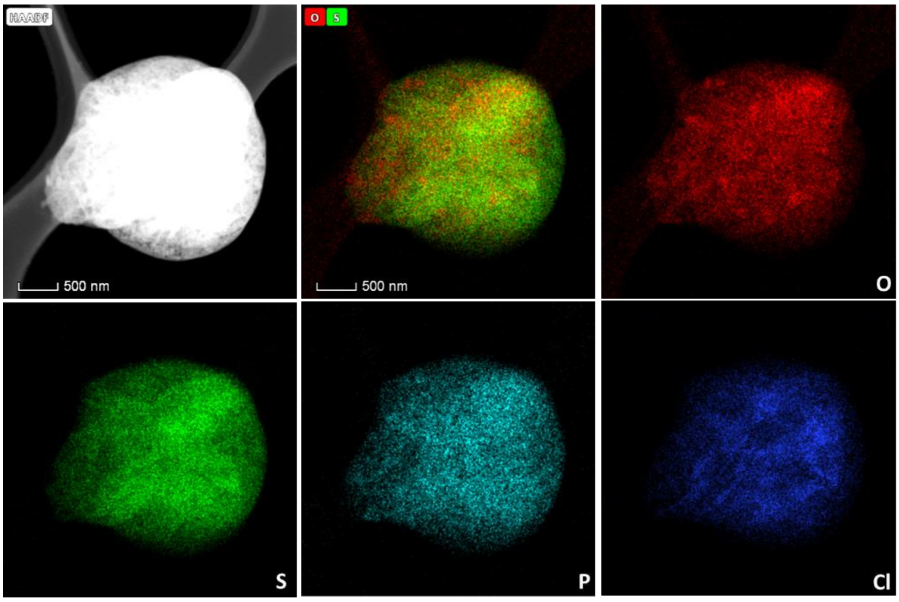

Figure S6. STEM-HADDF and EDS images of LPSC-O. No obvious shell structure was observed in LPSC-O, and O shows homogeneous distribution, indicating that O is successfully substituted for the S site.

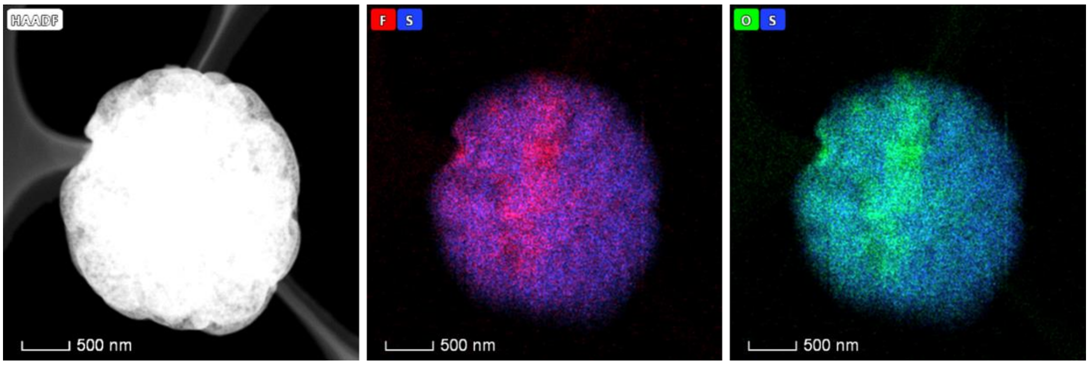

Figure S7. STEM-HADDF and EDS images of LPSC-$OF_{0.15}$. No obvious shell structure was observed in LPSC-$OF_{0.15}$, and O and F show homogeneous distribution, indicating that O is successfully substituted for the S site, and F is replaced the Cl site.

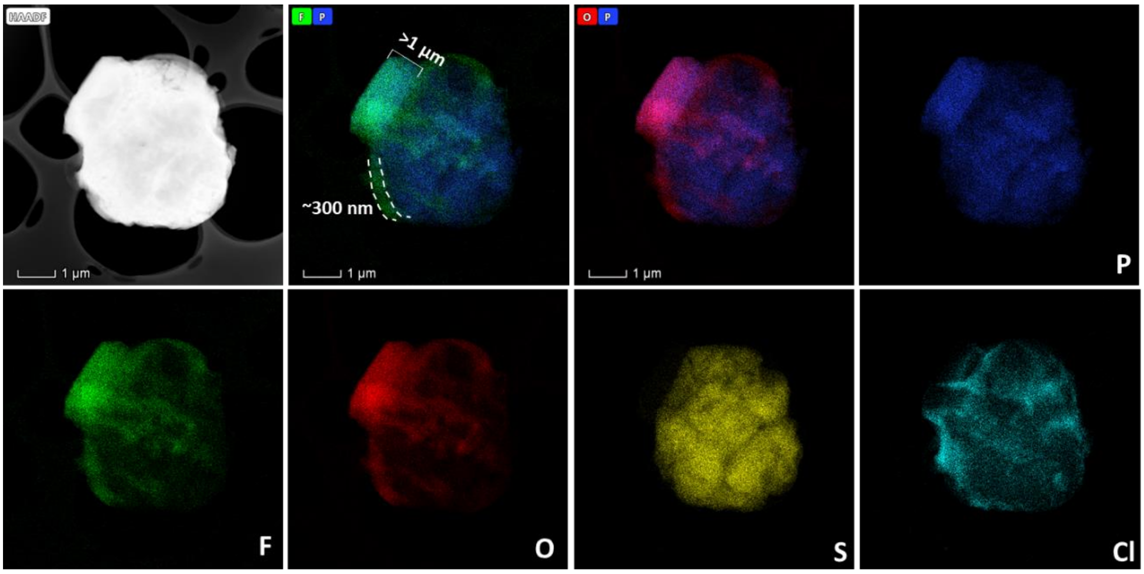

Figure S8. STEM-HADDF and EDS images of LPSC-$OF_{0.35}$. Apparently, increasing the proportion of F resulted in a thicker nanoshell and even micron-scale aggregation.

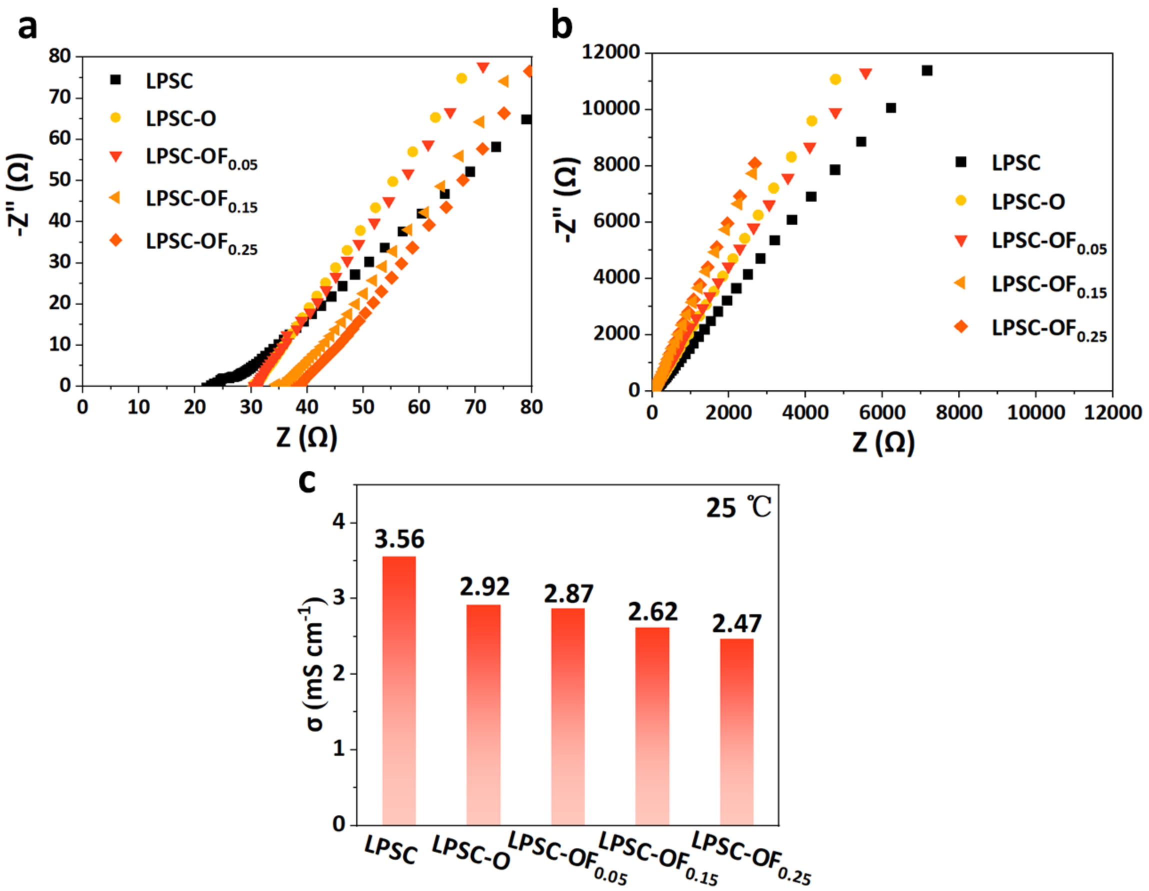

Figure S9. EIS of selected frequencies for measurements in SS|SE|SS cells at 25°C: (a) high frequency and (b) low frequency. (c) Ionic conductivity of SEs. The steep linear spike at low frequencies indicates that the as-synthesized LPSC-O_xF_y argyrodites are ionic conductors. The incomplete semicircles indicate a small grain boundary resistance, a typical feature of sulfide-based SEs that is favorable for battery assembly. However, it is hard to distinguish the grain boundary and bulk contribution based on these measured impedance spectra. Because the grain-boundary/bulk resistance cannot be clearly detected, it is hard to fit the Nyquist plots. The total ionic conductivity is thus calculated from the local minimal resistance at the intersection between the impedance spectrum and the x-axis.

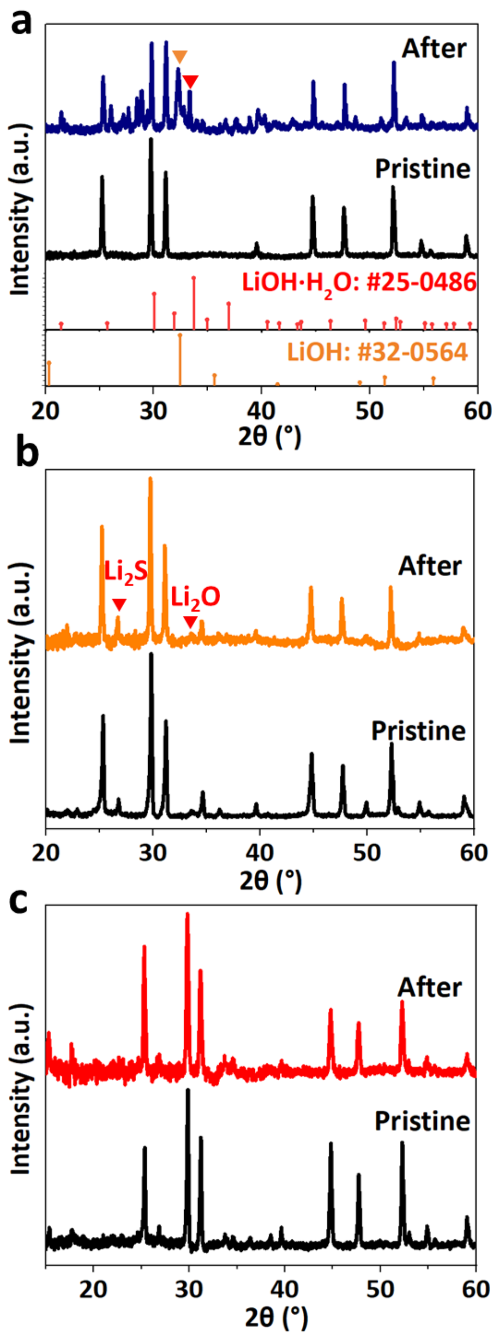

Figure S10. Contrast XRD patterns of pristine and post-annealed samples after exposure to air. (a) LPSC, (c) LPSC-O and (c) LPSC-$OF_{0.25}$.

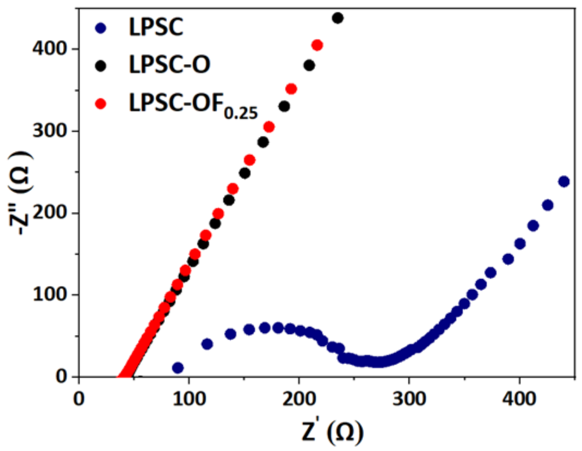

Figure S11. EIS of solid electrolytes after post-annealing.

Table S1. Air stability of LPSC-O_xF_y and previously reported solid electrolytes.

Ref.	Electrolyte	Relative humidity (%)	Ionic after exposed (mS cm^{-1})	Retention rate (%)
Our	**LPSC-$OF_{0.25}$**	**35±2**	**2.12**	**86**
	**LPSC-O**		**2.04**	**70**
	**LPSC**		**0.52**	**15**
[1]	gc-$Li_{3.2}P_{0.8}Sn_{0.2}S_4$	20	0.39	32
		5	1.03	85
[2]	Li_3InCl_6	30	0.8	52
[3]	$Li_3Y_{0.2}In_{0.8}Cl_6$	3-5	1.05	85
[4]	$Li_{3.85}Sn_{0.85}Sb_{0.15}S_4$	~0	0.41	48
[5]	LPSC@Li_2CO_3	17	~0.2	54

[1] F. Zhao, S. H. Alahakoon, K. Adair, S. Zhang, W. Xia, W. Li, C. Yu, R. Feng, Y. Hu, J. Liang, X. Lin, Y. Zhao, X. Yang, T.-K. Sham, H. Huang, L. Zhang, S. Zhao, S. Lu, Y. Huang, X. Sun, *Adv. Mater.* **2021**, *33*, 2006577.

[2] X. Li, J. Liang, N. Chen, J. Luo, K. R. Adair, C. Wang, M. N. Banis, T.-K. Sham, L. Zhang, S. Zhao, S. Lu, H. Huang, R. Li, X. Sun, *Angew. Chem. Int. Ed.* **2019**, *131*, 16579-16584.

[3] X. Li, J. Liang, K. R. Adair, J. Li, W. Li, F. Zhao, Y. Hu, T.-K. Sham, L. Zhang, S. Zhao, S. Lu, H. Huang, R. Li, N. Chen, X. Sun, *Nano Lett.* **2020**, *20*, 4384-4392.

[4] H. Kwak, K. H. Park, D. Han, K.-W. Nam, H. Kim, Y. S. Jung, *J. Power Sources* **2020**, *446*, 227338.

[5] X. Zhang, X. Li, S. Weng, S. Wu, Q. Liu, M. Cao, Y. Li, Z. Wang, L. Zhu, R. Xiao, D. Su, X. Yu, H. Li, L. Chen, Z. Wang, X. Wang, *Energy Environ. Sci.* **2023**, *16*, 1091-1099.

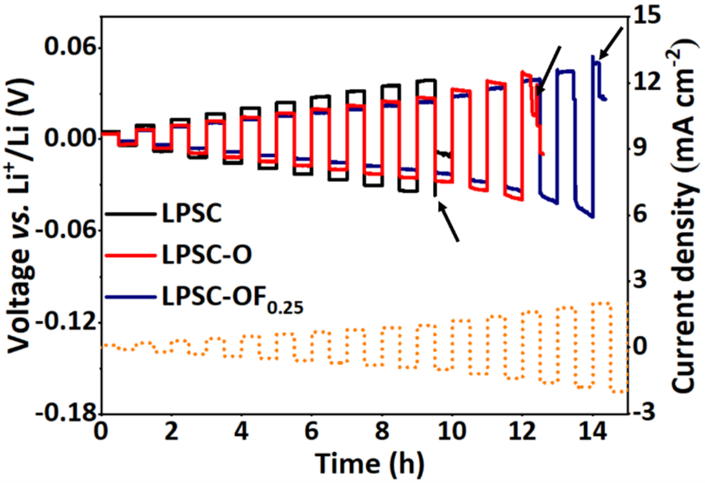

Figure S12. Voltage (solid lines) and current (dotted lines) curves with time for LPSC, LPSC-O and LPSC-$OF_{0.25}$. The observation from the figure clearly indicates that the symmetrical cell with the LPSC-$OF_{0.25}$ electrolyte exhibits a lower overpotential than those with LPSC and LPSC-O electrolytes. This seems contradictory, considering that the ionic conductivity of LPSC-$OF_{0.25}$ is slightly lower than those of LPSC and LPSC-O. However, the overpotential is determined by the overall impedance of the cell, which consists of both the bulk resistance of the electrolyte (directly linked to the ionic conductivity) and the interfacial resistance. In general, the interfacial resistance significantly outweighs the bulk resistance of the electrolyte, making it the primary factor in determining the value of overpotential. The symmetrical cell with the LPSC-$OF_{0.25}$ electrolyte demonstrates a lower interfacial resistance (Figure S15), and consequently, a reduced overpotential.

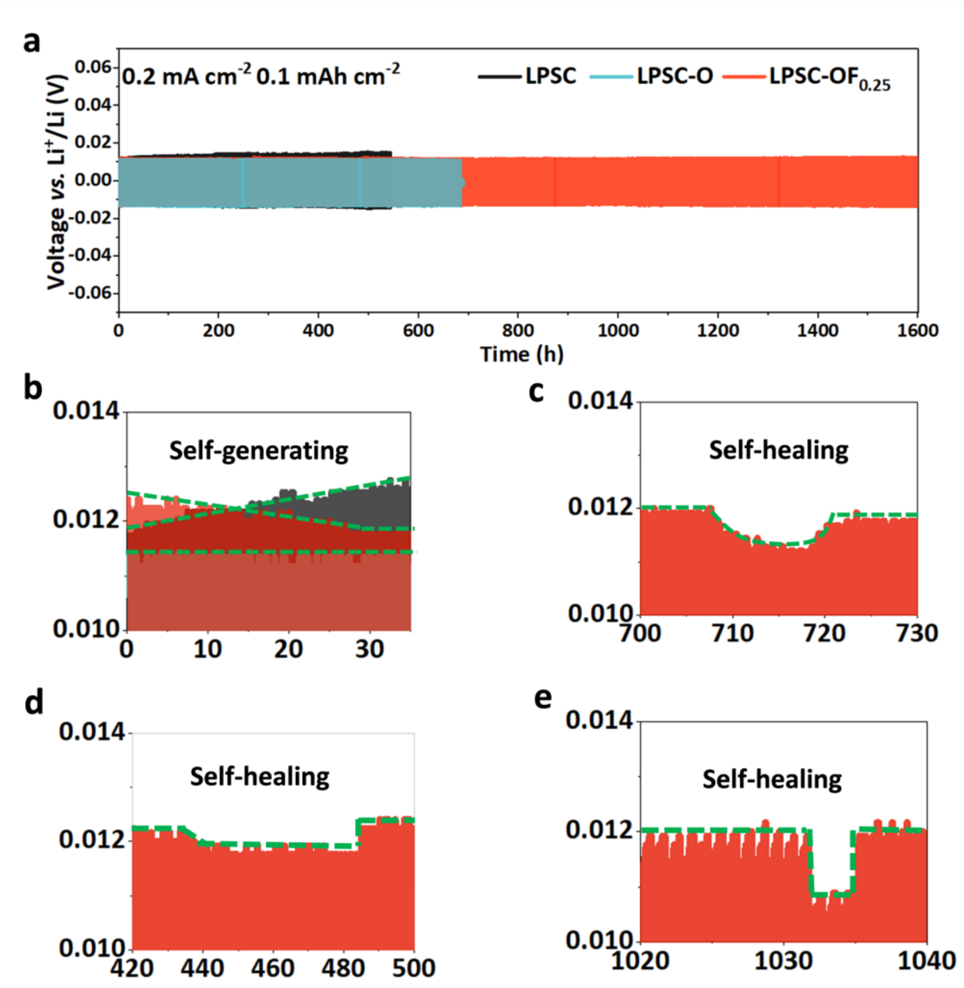

Figure S13. (a) Long-term Li plating/stripping cycling at 0.2 mA cm^{-2} with 0.1 mAh cm^{-2} for LPSC, LPSC-O and LPSC-OF$_{0.25}$. (b-d) Amplified curves of Li plating/stripping from (a).

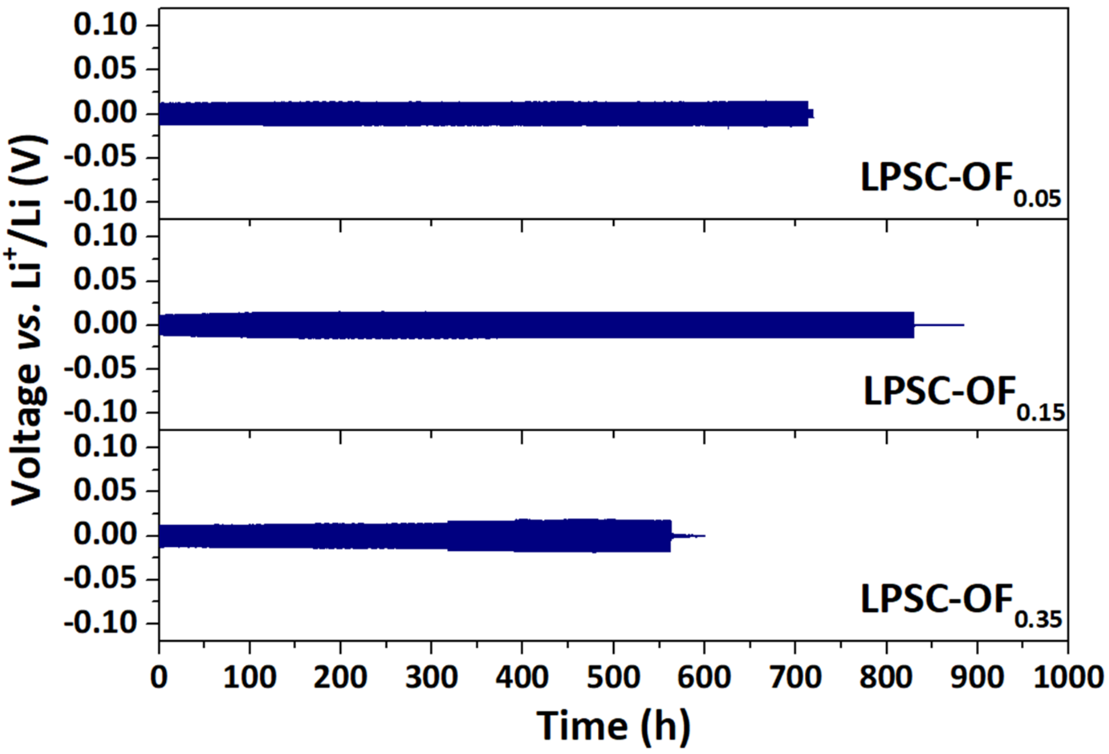

Figure S14. Long-term Li plating/stripping cycling at 0.2 mA cm^{-2} with 0.1 mAh cm^{-2} for LPSC-OF$_{0.05}$, LPSC-OF$_{0.15}$ and LPSC-OF$_{0.35}$.

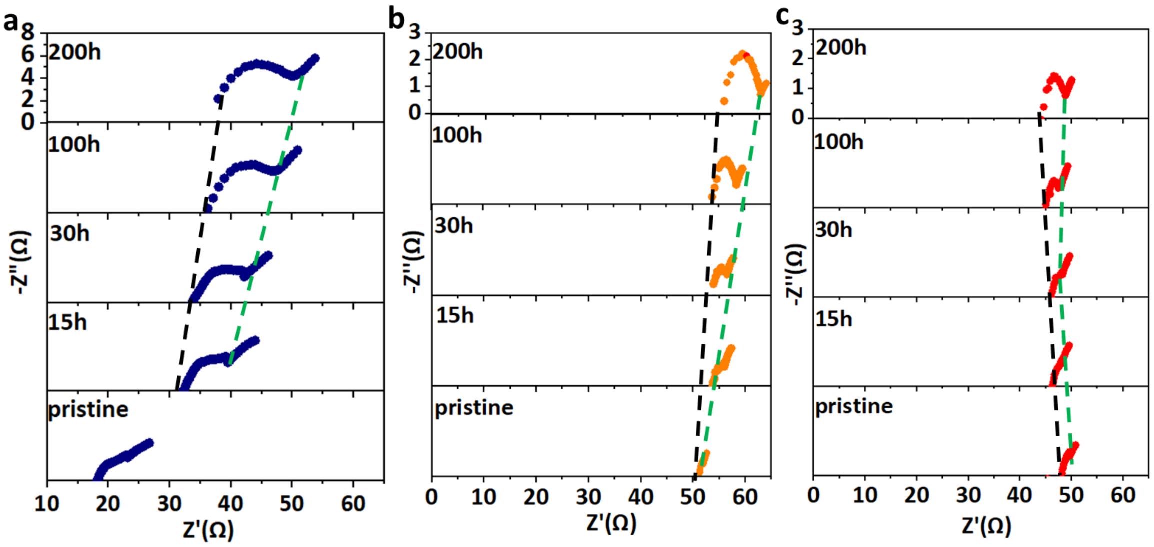

Figure S15. In-situ EIS measurements on the Li symmetric cells with (a) LPSC, (b) LPSC-O, and (d) LPSC-$OF_{0.25}$ as solid electrolytes.

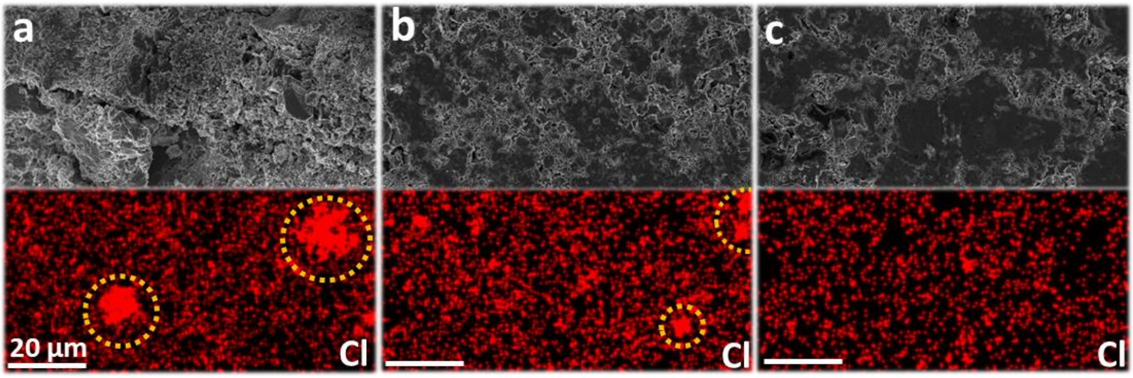

Figure S16. SEM: (b) LPSC, (c) LPSC-O, and (d) LPSC-$OF_{0.25}$ surface and the corresponding EDS of Cl element after cycles. The EDS mapping of the surface further to verify the above result; the decomposition products (chloride) in the LPSC-$OF_{0.25}$ does not show any significant clustering, different from large aggregates in LPSC and some small clusters in LPSC-O.

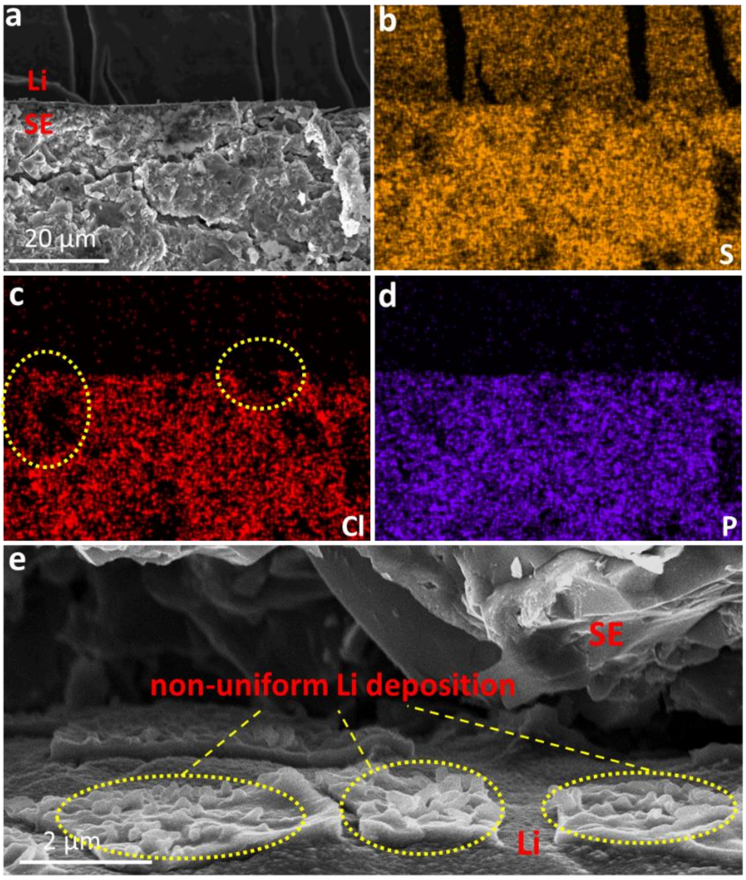

Figure S17. Cross-sectional SEM and EDS images for the Li|LPSC interface (a-d) and Li anode (e) after cycles. Through the cross-section diagram in Li|LPSC, the chloride distribution at the interface is irregular, and the sulfide has obvious diffusion. This is most likely caused by LPSC inability to form a stable and uniform SEI with lithium metal to prevent S continuous reaction and diffusion. On the contrast, the interface of Li|LPSC-$OF_{0.25}$ is much more complete and evener, and no significant bumpy and diffusion was found (Figure 3c). In addition, a significant amount of non-uniform lithium deposition was observed at the interface, and distinct columnar structures resembling dendrite precursors were identified (Figure 3e).

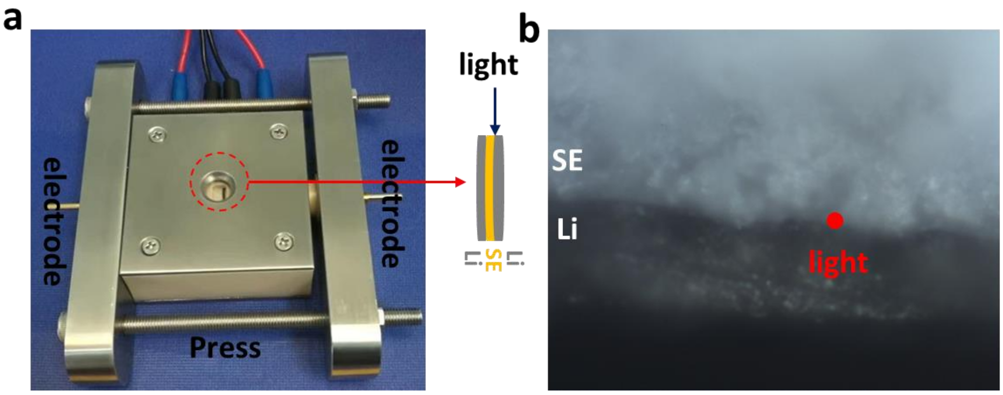

Figure S18. (a) Drawing of the *in situ* Raman mold. (b) Image of laser focusing area on the Li|LPSC-$OF_{0.25}$ interface.

Table S2. Stability of LPSC-$OF_{0.25}$ to lithium metal compared with the most advanced previously reported polymer, inorganic, and composite solid electrolytes.

Ref.	Solid electrolyte	Current density (mA cm^{-2})	Time (h)
**Our work**	**LPSC-$OF_{0.25}$**	**1**	**1270**
[1]	LiTFSI-PEO-AlF_3	0.1	1200
		0.2	560
[2]	$Li_{5.7}PS_{4.7}Cl_{1.3}$	0.5	1000
[3]	Li_6PS_5Cl-PEG-DME-LiFSI	0.1	500
[4]	Li_6PS_5Cl-PEG-DME	0.5	1000
[5]	$InCl_3$-$Li_{6.4}La_3Zr_{1.4}Ta_{0.6}O_{12}$ (LLZTO)	0.45	1000
[6]	W/LLZTO	1	200
[7]	$Li_{6.5}La_3Zr_{1.5}Ta_{0.5}O_{12}$	0.2	160
[8]	SnF_2-LLZTO	1	300
[9]	3D-LLZO	0.5	500
[10]	$Li_6PS_5Cl/Li_3YCl_6/Li_6PS_5Cl$	0.13	500
[11]	PEO-2,2'-bithiophene (PT)	0.05	720
	PT-PEO-PT		
[12]	$Li_7La_3Zr_{0.4}Hf_{0.4}Sn_{0.4}Sc_{0.4}Ta_{0.4}O_{12}$	0.2	400
[13]	Polyimide (PI)-(PEO/LiTFSI)	0.1	1000

[1] J. Hu, C. Lai, K. Chen, Q. Wu, Y. Gu, C. Wu, C. Li, *Nat. Commun.* **2022**, *13*, 7914.

[2] D. Zeng, J. Yao, L. Zhang, R. Xu, S. Wang, X. Yan, C. Yu, L. Wang, *Nat. Commun.* **2022**, *13*, 1909.

[3] H. Huo, M. Jiang, B. Mogwitz, J. Sann, Y. Yusim, T.-T. Zuo, Y. Moryson, P. Minnmann, F. H. Richter, C. V. Singh, J. JanekHuo, *Angew. Chem. Int. Ed.* **2023**, *62*, 202218044.

[4] X. Yang, X. Gao, M. Jiang, J. Luo, J. Yan, J. Fu, H. Duan, S. Zhao, Y. Tang, R. Yang, R. Li, J. Wang, H. Huang, C. V. Singh, X. Sun, *Angew. Chem. Int. Ed.* **2023**, *135*, 202215680.

[5] J. Leng, H. Liang, H. Wang, Z. Xiao, S. Wang, Z. Zhang, Z. Tang, *Nano Energy* **2022**, *101*, 107603.

[6] V. Raj, V. Venturi, V. R. Kankanallu, B. Kuiri, V. Viswanathan, N. P. B. Aetukuri, *Nat. Mater.* **2022**, *21*, 1050-1056.

[7] S. Kim, J.-S. Kim, L. Miara, Y. Wang, S.-K. Jung, S. Y. Park, Z. Song, H. Kim, M. Badding, J. Chang, V. Roev, G. Yoon, R. Kim, J.-H. Kim, K. Yoon, D. Im, K. Kang, *Nat. Commun.* **2022**, *13*, 1883.

[8] K. Lee, S. Han, J. Lee, S. Lee, J. Kim, Y. Ko, S. Kim, K. Yoon, J.-H. Song, J. H. Noh, K. Kang, *ACS Energy Lett.* **2021**, *7*, 381-389.

[9] R. Xu, F. Liu, Y. Ye, H. Chen, R. R. Yang, Y. Ma, W. Huang, J. Wan, Y. Cui, *Adv. Mater.* **2021**, *33*, 2104009.

[10] C. Wang, J. Liang, J. Luo, J. Liu, X. Li, F. Zhao, R. Li, H. Huang, S. Zhao, L. Zhang, J. Wang, X. Sun, *Sci. Adv.* **2021**, *7*, eabh1896.

[11] J. Zheng, C. Sun, Z. Wang, S. Liu, B. An, Z. Sun, F. Li, *Angew. Chem. Int. Ed.* **2021**, *60*, 18448-18453.

[12] S.-K. Jung, H. Gwon, H. Kim, G. Yoon, D. Shin, J. Hong, C. Jung, J.-S. Kim, *Nat. Commun.* **2022**, *13*, 7638.

[13] J. Wan, J. Xie, X. Kong, Z. Liu, K. Liu, F. Shi, A. Pei, H. Chen, W. Chen, J. Chen, X. Zhang, L. Zong, J. Wang, L.-Q. Chen, J. Qin, Y. Cui, *Nat. Nanotechnol.* **2019**, *14*, 705-711.

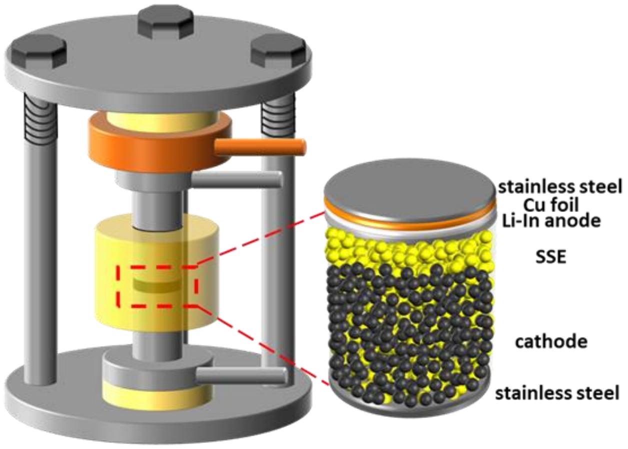

Figure S19. Schematic diagram of a pressurized cell.

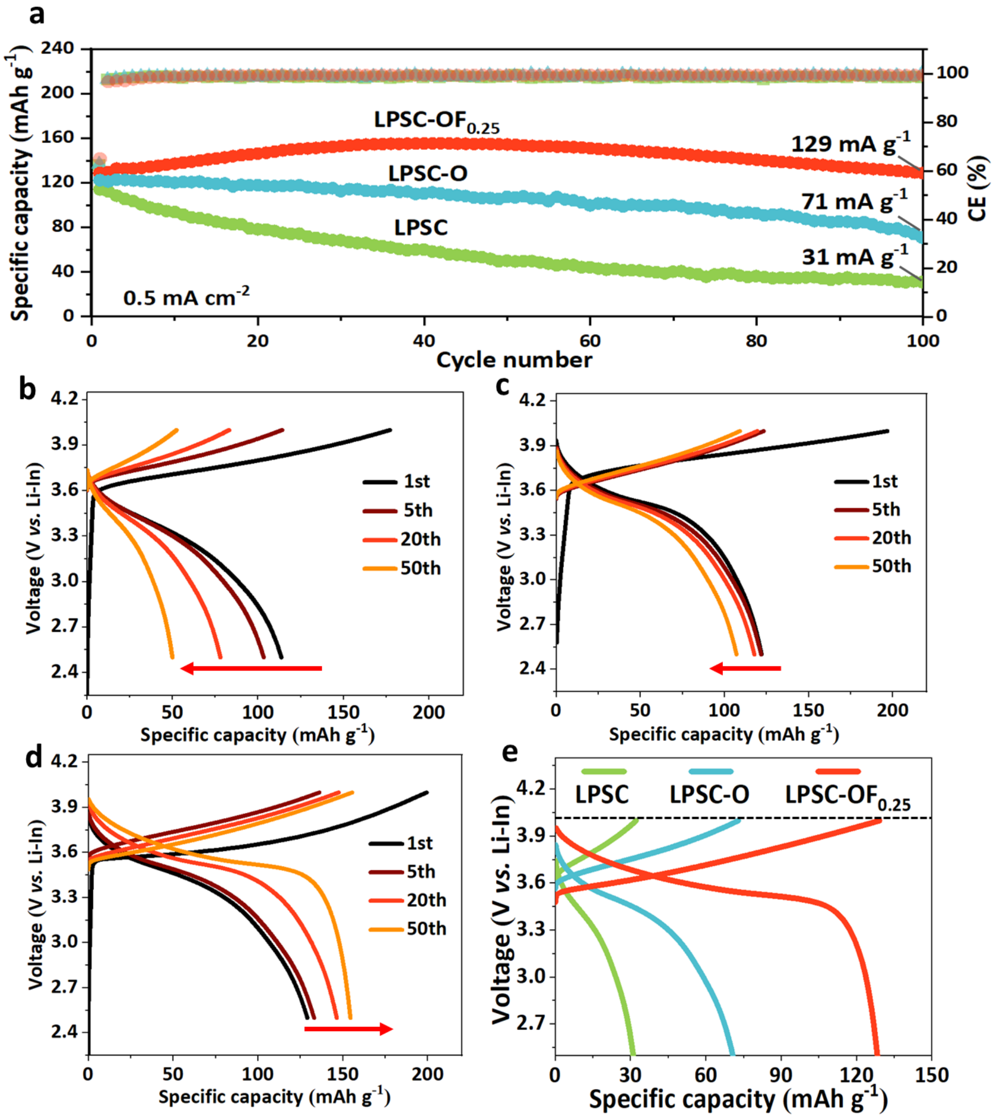

Figure S20. (a) Discharge capacity and coulombic efficiency, and (b-e) corresponding charge/discharge profiles at 0.5 mAh cm^{-2} within the voltage of 2.5-4 V (*vs.* Li-In). Charge/discharge profiles for Li-In|SE|LCO cells with (b) LPSC, (c) LPSC-O, and (d) LPSC-$OF_{0.25}$ as the solid electrolyte and ion conductive additive in composite cathodes. Charge/discharge profiles for Li-In|SE|LCO cells after 100 cycles.

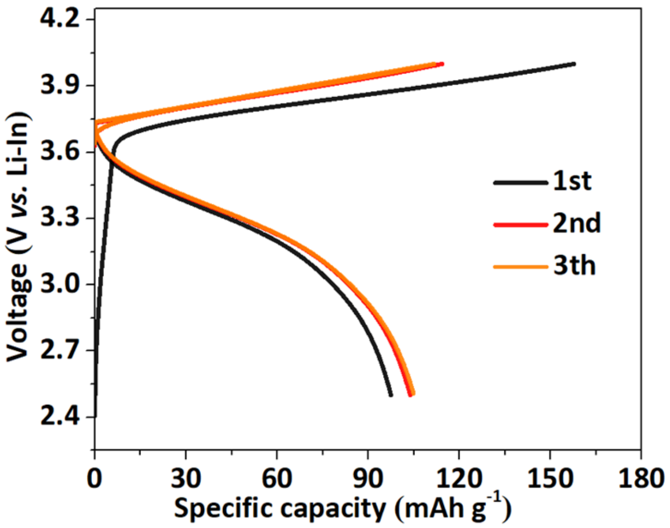

Figure S21. Charge/discharge curves at 0.5 mAh cm^{-2} within the voltage range of 2.5-4 V (*vs.* Li-In) for Li-In|SE|LCO cells with LPSC-$OF_{0.25}$ as the solid electrolyte and ion conductive additive in composite cathodes (25℃).

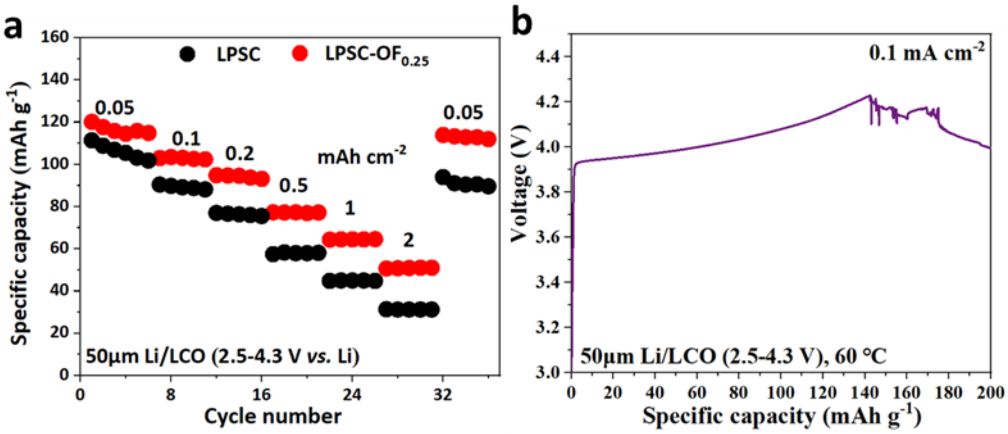

Figure S22. (a) Rate performance for Li|LPSC-$OF_{0.25}$|LCO cells within the voltage of 2.5-4.3 V (10 MPa). (b) Charge/discharge profiles at 0.1 mAh cm^{-2} within the voltage of 2.5-4.3 V for Li|LPSC-$OF_{0.25}$|LCO cells (50 MPa).

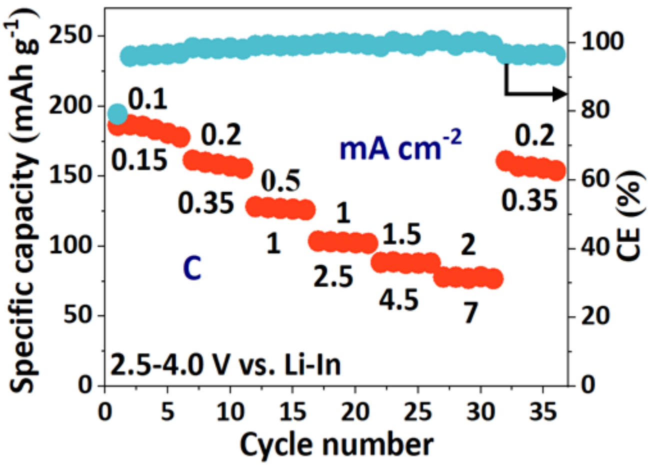

Figure S23. Rate performance and Coulombic efficiency for Li-In|LPSC-$OF_{0.25}$|LCO cells within the voltage of 2.5-4 V (*vs.* Li-In). In order to accurately obtain more realistic rate performance, we used the current density (mA cm^{-2}) divided by the areal capacity (mAh cm^{-2}) to obtain the C-rate (h^{-1}).

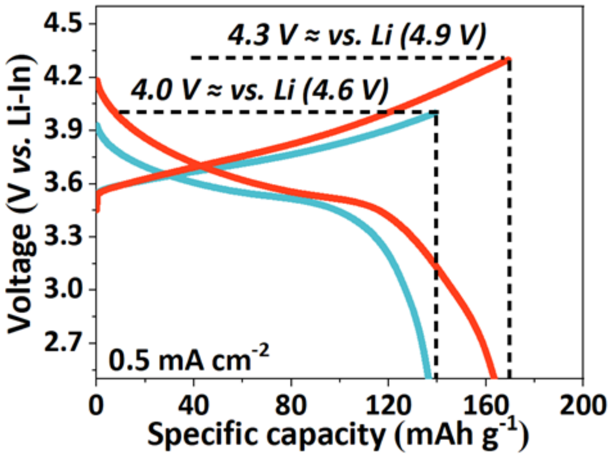

Figure S24. Charge/discharge profiles for Li-In|LPSC-$OF_{0.25}$|LCO cells within different voltage ranges (2.5-4 V and 2.5-4.3 V *vs.* Li-In) under 0.5 mA cm^{-2}. When considering the typical LCO behavior during discharge, the change of discharge curves to steep slope is around 3.8 V vs. Li/Li^+ while the value in Figure S22 is about 3.45 V vs. Li-In, so we can translate to an actual charging cutoff potential of about 4.65 V vs. Li/Li^+.

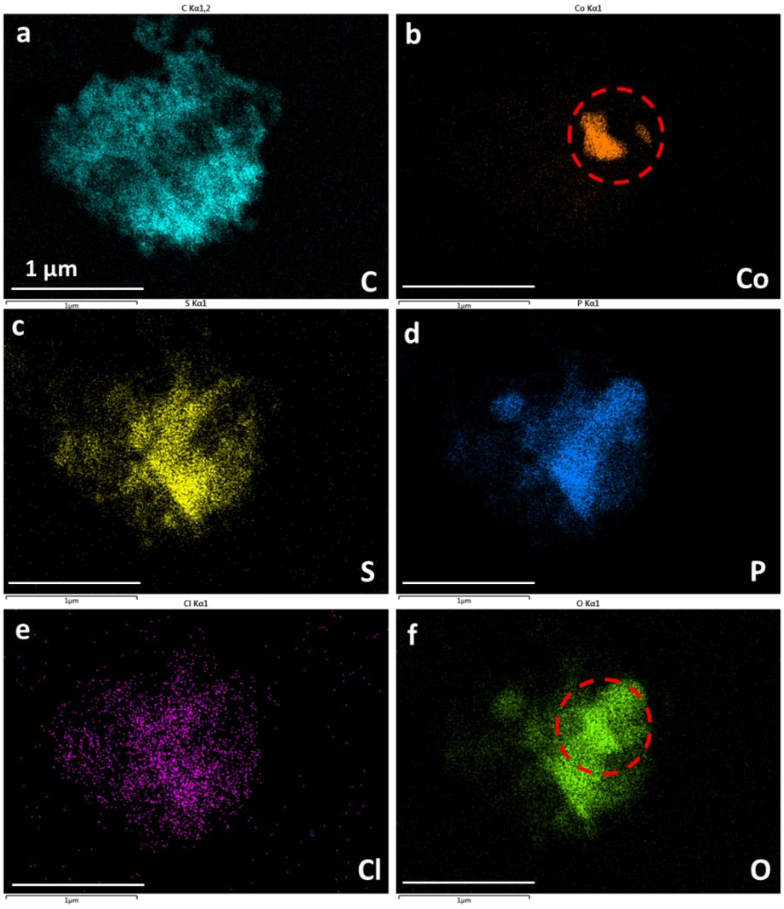

Figure S25. EDS images of the as-prepared composite cathode powder after 50 cycles (with LPSC-$OF_{0.25}$ as the solid electrolyte and ion conductive additive in composite cathodes).

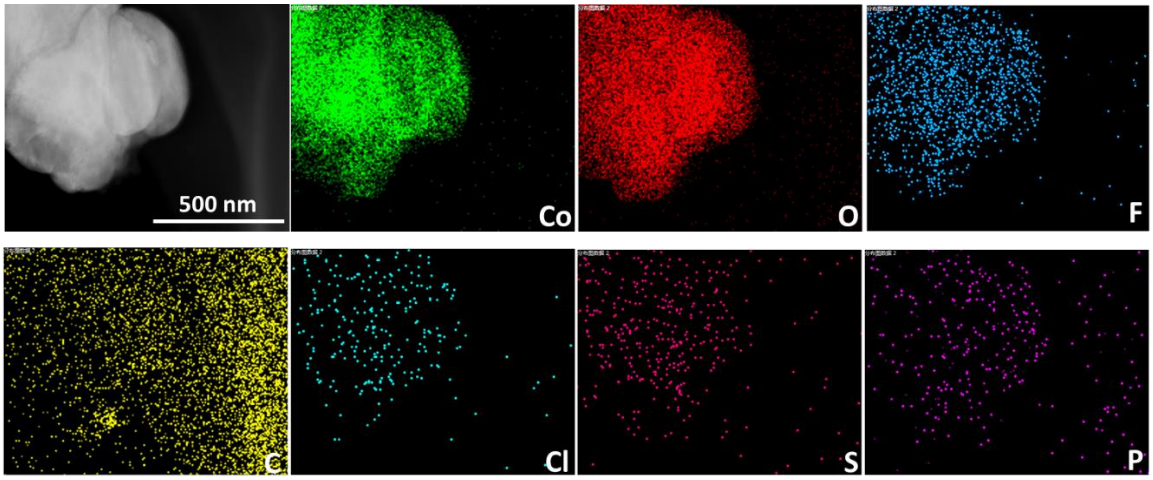

Figure S26. STEM-HAADF and EDS images of the divested LCO after 50 cycles (with LPSC-$OF_{0.25}$ as the solid electrolyte and ion conductive additive in composite cathodes).

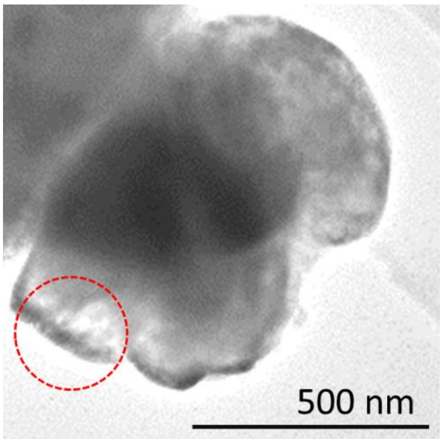

Figure S27. Cry-TEM image of the divested LCO from composite cathodes after 50 cycles (with LPSC-$OF_{0.25}$ as the solid electrolyte and ion conductive additive in composite cathodes).

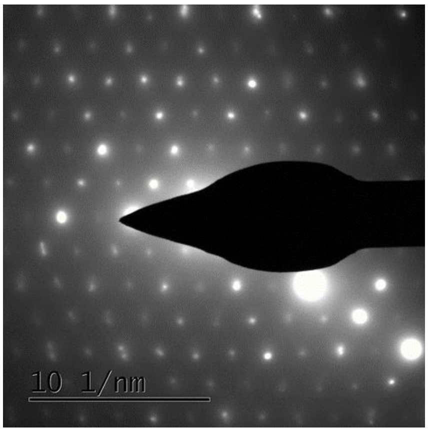

Figure S28. SAED image of the pristine LCO.

Table S3. Cycling stability of LPSC-$OF_{0.25}$|LCO cells compared with the most advanced previously reported full cells with polymer, inorganic, and composite solid electrolytes matched with LFP, LCO, or NCM cathodes.

Ref.	Solid electrolyte	Cathode active materials	Current density (mA cm^{-2})	Cycle number (n)	retention rate (%)
**Our work**	**LPSC-$OF_{0.25}$**	**$LiCoO_2$ (LCO)**	**2**	**500**	**81**
				**800**	**60**
[1]	Li_6PS_5Cl-PEGDME-LiFSI	$LiNi_{0.85}Co_{0.1}Mn_{0.05}O_2$ @ $LiNbO_3$ (NCM-85@LNO)	0.09	100	81.2
[2]	Li_6PS_5Cl-PEGDME	(Li_2ZrO_3) LZO@LCO	0.5	650	80
[3]	$Li_{6.5}La_3Zr_{1.5}Ta_{0.5}O_{12}$ (Ta–LLZO)	NCM811	1.6	100	87
[4]	SnF_2-LLZTO	$LiFePO_4$ (LFP)	1	600	-
[5]	$Li_6PS_5Cl/Li_3YCl_6/Li_6PS_5Cl$	$LiCoO_2$	0.13	50	85
[6]	$Li_7La_3Zr_{0.4}Hf_{0.4}Sn_{0.4}Sc_{0.4}Ta_{0.4}O_{12}$	$LiNi_{1/3}Co_{1/3}Mn_{1/3}O_2$ (NCM111)	0.8	700	92
[7]	FMC-ASPE-Li	NMC811	0.16	100	87
[8]	poly-DOL SP	LFP	0.75	700	~75
		$LiNi_{0.5}Mn_{0.3}Co_{0.2}O_2$ (NCN523)	0.07	100	90.7
[9]	$Li_{3-x}(OH_x)Cl_{0.9}F_{0.1}$	LFP	0.05	50	70.1
[10]	$Li_{10}GeP_2S_{12}$	PS-LPO-NMC811	0.178	250	80
[11]	PCEE	$LiNi_{0.83}Mn_{0.06}Co_{0.11}O_2$ (NMC-83)	0.24	250	80
[12]	LPSC-LZCLi_2ZrCl_6	single-crystal sc-NMC811	1.34	200	~95
		LCO	0.47	100	~90
[13]	LLZTO–PCE	LFP	0.045	300	65
[14]	PEO-Li_2S	LFP	0.1	700	89.2

[1] H. Huo, M. Jiang, B. Mogwitz, J. Sann, Y. Yusim, T.-T. Zuo, Y. Moryson, P. Minnmann, F. H. Richter, C. V. Singh, J. JanekHuo, *Angew. Chem. Int. Ed.* **2023**, *62*, 202218044.

[2] X. Yang, X. Gao, M. Jiang, J. Luo, J. Yan, J. Fu, H. Duan, S. Zhao, Y. Tang, R. Yang, R. Li, J. Wang, H. Huang, C. V. Singh, X. Sun, *Angew. Chem. Int. Ed.* **2023**, *135*, 202215680.

[3] S. Kim, J.-S. Kim, L. Miara, Y. Wang, S.-K. Jung, S. Y. Park, Z. Song, H. Kim, M. Badding, J. Chang, V. Roev, G. Yoon, R. Kim, J.-H. Kim, K. Yoon, D. Im, K. Kang, *Nat. Commun.* **2022**, *13*, 1883.

[4] K. Lee, S. Han, J. Lee, S. Lee, J. Kim, Y. Ko, S. Kim, K. Yoon, J.-H. Song, J. H. Noh, K. Kang, *ACS Energy Lett.* **2021**, *7*, 381-389.

[5] C. Wang, J. Liang, J. Luo, J. Liu, X. Li, F. Zhao, R. Li, H. Huang, S. Zhao, L. Zhang, J. Wang, X. Sun, *Sci. Adv.* **2021**, *7*, eabh1896.

[6] S.-K. Jung, H. Gwon, H. Kim, G. Yoon, D. Shin, J. Hong, C. Jung, J.-S. Kim, *Nat. Commun.* **2022**, *13*, 7638.

[7] Y. Su, X. Rong, A. Gao, Y. Liu, J. Li, M. Mao, X. Qi, G. Chai, Q. Zhang, L. Suo, L. Gu, H. Li, X. Huang, L. Chen, B. Liu, Y.-S. Hu, *Nat. Commun.* **2022**, *13*, 4181.

[8] Q. Zhao, X. Liu, S. Stalin, K. Khan, L. A. Archer, *Nat. Energy* **2019**, *4*, 365-373.

[9] W. Feng, L. Zhu, X. Dong, Y. Wang, Y. Xia, F. Wang, *Adv. Mater.* **2023**, *35*, 2210365.

[10] J. Liang, Y. Zhu, X. Li, J. Luo, S. Deng, Y. Zhao, Y. Sun, D. Wu, Y. Hu, W. Li, T.-K. Sham, R. Li, M. Gu, X. Sun, *Nat. Commun.* **2023**, *14*, 146.

[11] J. Han, M. J. Lee, K. Lee, Y. J. Lee, S. H. Kwon, J. H. Min, E. Lee, W. Lee, S. W.Lee, B. J. Kim, *Adv Mater* **2023**, *35*, 2205194.

[12] K. Wang, Q. Ren, Z. Gu, C. Duan, J. Wang, F. Zhu, Y. Fu, J. Hao, J. Zhu, L. He, C.-W. Wang, Y. Lu, J. Ma, C. Ma, *Nat. Commun.* **2021**, *12*, 4410.

[13] S. Chen, J. Zhang, L. Nie, X. Hu, Y. Huang, Y. Yu, W. Liu, *Adv. Mater.* **2021**, *33*, 2002325.

[14] R. Fang, B. Xu, N. S. Grundish, Y. Xia, Y. Li, C. Lu, Y. Liu, N. Wu, J. B. Goodenough, *Angew. Chem. Int. Ed.* **2021**, *133*, 17842-17847.
